# Search for new physics in same-sign dilepton events in proton–proton collisions at $\sqrt{s} = 13\,\text {TeV}$

**DOI:** 10.1140/epjc/s10052-016-4261-z

**Published:** 2016-08-05

**Authors:** V. Khachatryan, A. M. Sirunyan, A. Tumasyan, W. Adam, E. Asilar, T. Bergauer, J. Brandstetter, E. Brondolin, M. Dragicevic, J. Erö, M. Flechl, M. Friedl, R. Frühwirth, V. M. Ghete, C. Hartl, N. Hörmann, J. Hrubec, M. Jeitler, A. König, I. Krätschmer, D. Liko, T. Matsushita, I. Mikulec, D. Rabady, N. Rad, B. Rahbaran, H. Rohringer, J. Schieck, J. Strauss, W. Treberer-Treberspurg, W. Waltenberger, C.-E. Wulz, V. Mossolov, N. Shumeiko, J. Suarez Gonzalez, S. Alderweireldt, E. A. De Wolf, X. Janssen, J. Lauwers, M. Van De Klundert, H. Van Haevermaet, P. Van Mechelen, N. Van Remortel, A. Van Spilbeeck, S. Abu Zeid, F. Blekman, J. D’Hondt, N. Daci, I. De Bruyn, K. Deroover, N. Heracleous, S. Lowette, S. Moortgat, L. Moreels, A. Olbrechts, Q. Python, S. Tavernier, W. Van Doninck, P. Van Mulders, I. Van Parijs, H. Brun, C. Caillol, B. Clerbaux, G. De Lentdecker, H. Delannoy, G. Fasanella, L. Favart, R. Goldouzian, A. Grebenyuk, G. Karapostoli, T. Lenzi, A. Léonard, J. Luetic, T. Maerschalk, A. Marinov, A. Randle-Conde, T. Seva, C. Vander Velde, P. Vanlaer, R. Yonamine, F. Zenoni, F. Zhang, A. Cimmino, T. Cornelis, D. Dobur, A. Fagot, G. Garcia, M. Gul, D. Poyraz, S. Salva, R. Schöfbeck, M. Tytgat, W. Van Driessche, E. Yazgan, N. Zaganidis, H. Bakhshiansohi, C. Beluffi, O. Bondu, S. Brochet, G. Bruno, A. Caudron, L. Ceard, S. De Visscher, C. Delaere, M. Delcourt, L. Forthomme, B. Francois, A. Giammanco, A. Jafari, P. Jez, M. Komm, V. Lemaitre, A. Magitteri, A. Mertens, M. Musich, C. Nuttens, K. Piotrzkowski, L. Quertenmont, M. Selvaggi, M. Vidal Marono, S. Wertz, N. Beliy, W. L. Aldá Júnior, F. L. Alves, G. A. Alves, L. Brito, C. Hensel, A. Moraes, M. E. Pol, P. Rebello Teles, E. Belchior Batista Das Chagas, W. Carvalho, J. Chinellato, A. Custódio, E. M. Da Costa, G. G. Da Silveira, D. De Jesus Damiao, C. De Oliveira Martins, S. Fonseca De Souza, L. M. Huertas Guativa, H. Malbouisson, D. Matos Figueiredo, C. Mora Herrera, L. Mundim, H. Nogima, W. L. Prado Da Silva, A. Santoro, A. Sznajder, E. J. Tonelli Manganote, A. Vilela Pereira, S. Ahuja, C. A. Bernardes, S. Dogra, T. R. Fernandez Perez Tomei, E. M. Gregores, P. G. Mercadante, C. S. Moon, S. F. Novaes, Sandra S. Padula, D. Romero Abad, J. C. Ruiz Vargas, A. Aleksandrov, R. Hadjiiska, P. Iaydjiev, M. Rodozov, S. Stoykova, G. Sultanov, M. Vutova, A. Dimitrov, I. Glushkov, L. Litov, B. Pavlov, P. Petkov, W. Fang, M. Ahmad, J. G. Bian, G. M. Chen, H. S. Chen, M. Chen, Y. Chen, T. Cheng, C. H. Jiang, D. Leggat, Z. Liu, F. Romeo, S. M. Shaheen, A. Spiezia, J. Tao, C. Wang, Z. Wang, H. Zhang, J. Zhao, Y. Ban, Q. Li, S. Liu, Y. Mao, S. J. Qian, D. Wang, Z. Xu, C. Avila, A. Cabrera, L. F. Chaparro Sierra, C. Florez, J. P. Gomez, C. F. González Hernández, J. D. Ruiz Alvarez, J. C. Sanabria, N. Godinovic, D. Lelas, I. Puljak, P. M. Ribeiro Cipriano, Z. Antunovic, M. Kovac, V. Brigljevic, D. Ferencek, K. Kadija, S. Micanovic, L. Sudic, A. Attikis, G. Mavromanolakis, J. Mousa, C. Nicolaou, F. Ptochos, P. A. Razis, H. Rykaczewski, M. Finger, M. Finger, E. Carrera Jarrin, S. Elgammal, A. Mohamed, Y. Mohammed, E. Salama, B. Calpas, M. Kadastik, M. Murumaa, L. Perrini, M. Raidal, A. Tiko, C. Veelken, P. Eerola, J. Pekkanen, M. Voutilainen, J. Härkönen, V. Karimäki, R. Kinnunen, T. Lampén, K. Lassila-Perini, S. Lehti, T. Lindén, P. Luukka, T. Peltola, J. Tuominiemi, E. Tuovinen, L. Wendland, J. Talvitie, T. Tuuva, M. Besancon, F. Couderc, M. Dejardin, D. Denegri, B. Fabbro, J. L. Faure, C. Favaro, F. Ferri, S. Ganjour, S. Ghosh, A. Givernaud, P. Gras, G. Hamel de Monchenault, P. Jarry, I. Kucher, E. Locci, M. Machet, J. Malcles, J. Rander, A. Rosowsky, M. Titov, A. Zghiche, A. Abdulsalam, I. Antropov, S. Baffioni, F. Beaudette, P. Busson, L. Cadamuro, E. Chapon, C. Charlot, O. Davignon, R. Granier de Cassagnac, M. Jo, S. Lisniak, P. Miné, I. N. Naranjo, M. Nguyen, C. Ochando, G. Ortona, P. Paganini, P. Pigard, S. Regnard, R. Salerno, Y. Sirois, T. Strebler, Y. Yilmaz, A. Zabi, J.-L. Agram, J. Andrea, A. Aubin, D. Bloch, J.-M. Brom, M. Buttignol, E. C. Chabert, N. Chanon, C. Collard, E. Conte, X. Coubez, J.-C. Fontaine, D. Gelé, U. Goerlach, A.-C. Le Bihan, J. A. Merlin, K. Skovpen, P. Van Hove, S. Gadrat, S. Beauceron, C. Bernet, G. Boudoul, E. Bouvier, C. A. Carrillo Montoya, R. Chierici, D. Contardo, B. Courbon, P. Depasse, H. El Mamouni, J. Fan, J. Fay, S. Gascon, M. Gouzevitch, G. Grenier, B. Ille, F. Lagarde, I. B. Laktineh, M. Lethuillier, L. Mirabito, A. L. Pequegnot, S. Perries, A. Popov, D. Sabes, V. Sordini, M. Vander Donckt, P. Verdier, S. Viret, A. Khvedelidze, D. Lomidze, C. Autermann, S. Beranek, L. Feld, A. Heister, M. K. Kiesel, K. Klein, M. Lipinski, A. Ostapchuk, M. Preuten, F. Raupach, S. Schael, C. Schomakers, J. F. Schulte, J. Schulz, T. Verlage, H. Weber, V. Zhukov, M. Brodski, E. Dietz-Laursonn, D. Duchardt, M. Endres, M. Erdmann, S. Erdweg, T. Esch, R. Fischer, A. Güth, T. Hebbeker, C. Heidemann, K. Hoepfner, S. Knutzen, M. Merschmeyer, A. Meyer, P. Millet, S. Mukherjee, M. Olschewski, K. Padeken, P. Papacz, T. Pook, M. Radziej, H. Reithler, M. Rieger, F. Scheuch, L. Sonnenschein, D. Teyssier, S. Thüer, V. Cherepanov, Y. Erdogan, G. Flügge, W. Haj Ahmad, F. Hoehle, B. Kargoll, T. Kress, A. Künsken, J. Lingemann, A. Nehrkorn, A. Nowack, I. M. Nugent, C. Pistone, O. Pooth, A. Stahl, M. Aldaya Martin, C. Asawatangtrakuldee, I. Asin, K. Beernaert, O. Behnke, U. Behrens, A. A. Bin Anuar, K. Borras, A. Campbell, P. Connor, C. Contreras-Campana, F. Costanza, C. Diez Pardos, G. Dolinska, G. Eckerlin, D. Eckstein, E. Gallo, J. Garay Garcia, A. Geiser, A. Gizhko, J. M. Grados Luyando, P. Gunnellini, A. Harb, J. Hauk, M. Hempel, H. Jung, A. Kalogeropoulos, O. Karacheban, M. Kasemann, J. Keaveney, J. Kieseler, C. Kleinwort, I. Korol, W. Lange, A. Lelek, J. Leonard, K. Lipka, A. Lobanov, W. Lohmann, R. Mankel, I.-A. Melzer-Pellmann, A. B. Meyer, G. Mittag, J. Mnich, A. Mussgiller, E. Ntomari, D. Pitzl, R. Placakyte, A. Raspereza, B. Roland, M. Ö. Sahin, P. Saxena, T. Schoerner-Sadenius, C. Seitz, S. Spannagel, N. Stefaniuk, K. D. Trippkewitz, G. P. Van Onsem, R. Walsh, C. Wissing, V. Blobel, M. Centis Vignali, A. R. Draeger, T. Dreyer, E. Garutti, K. Goebel, D. Gonzalez, J. Haller, M. Hoffmann, A. Junkes, R. Klanner, R. Kogler, N. Kovalchuk, T. Lapsien, T. Lenz, I. Marchesini, D. Marconi, M. Meyer, M. Niedziela, D. Nowatschin, J. Ott, F. Pantaleo, T. Peiffer, A. Perieanu, J. Poehlsen, C. Sander, C. Scharf, P. Schleper, A. Schmidt, S. Schumann, J. Schwandt, H. Stadie, G. Steinbrück, F. M. Stober, M. Stöver, H. Tholen, D. Troendle, E. Usai, L. Vanelderen, A. Vanhoefer, B. Vormwald, C. Barth, C. Baus, J. Berger, E. Butz, T. Chwalek, F. Colombo, W. De Boer, A. Dierlamm, S. Fink, R. Friese, M. Giffels, A. Gilbert, D. Haitz, F. Hartmann, S. M. Heindl, U. Husemann, I. Katkov, P. Lobelle Pardo, B. Maier, H. Mildner, M. U. Mozer, T. Müller, Th. Müller, M. Plagge, G. Quast, K. Rabbertz, S. Röcker, F. Roscher, M. Schröder, G. Sieber, H. J. Simonis, R. Ulrich, J. Wagner-Kuhr, S. Wayand, M. Weber, T. Weiler, S. Williamson, C. Wöhrmann, R. Wolf, G. Anagnostou, G. Daskalakis, T. Geralis, V. A. Giakoumopoulou, A. Kyriakis, D. Loukas, I. Topsis-Giotis, A. Agapitos, S. Kesisoglou, A. Panagiotou, N. Saoulidou, E. Tziaferi, I. Evangelou, G. Flouris, C. Foudas, P. Kokkas, N. Loukas, N. Manthos, I. Papadopoulos, E. Paradas, N. Filipovic, G. Bencze, C. Hajdu, P. Hidas, D. Horvath, F. Sikler, V. Veszpremi, G. Vesztergombi, A. J. Zsigmond, N. Beni, S. Czellar, J. Karancsi, A. Makovec, J. Molnar, Z. Szillasi, M. Bartók, P. Raics, Z. L. Trocsanyi, B. Ujvari, S. Bahinipati, S. Choudhury, P. Mal, K. Mandal, A. Nayak, D. K. Sahoo, N. Sahoo, S. K. Swain, S. Bansal, S. B. Beri, V. Bhatnagar, R. Chawla, U. Bhawandeep, A. K. Kalsi, A. Kaur, M. Kaur, R. Kumar, A. Mehta, M. Mittal, J. B. Singh, G. Walia, Ashok Kumar, A. Bhardwaj, B. C. Choudhary, R. B. Garg, S. Keshri, A. Kumar, S. Malhotra, M. Naimuddin, N. Nishu, K. Ranjan, R. Sharma, V. Sharma, R. Bhattacharya, S. Bhattacharya, K. Chatterjee, S. Dey, S. Dutt, S. Dutta, S. Ghosh, N. Majumdar, A. Modak, K. Mondal, S. Mukhopadhyay, S. Nandan, A. Purohit, A. Roy, D. Roy, S. Roy Chowdhury, S. Sarkar, M. Sharan, S. Thakur, P. K. Behera, R. Chudasama, D. Dutta, V. Jha, V. Kumar, A. K. Mohanty, P. K. Netrakanti, L. M. Pant, P. Shukla, A. Topkar, S. Bhowmik, R. K. Dewanjee, S. Ganguly, S. Kumar, M. Maity, B. Parida, T. Sarkar, T. Aziz, S. Dugad, G. Kole, B. Mahakud, S. Mitra, G. B. Mohanty, N. Sur, B. Sutar, S. Banerjee, M. Guchait, Sa. Jain, G. Majumder, K. Mazumdar, N. Wickramage, S. Chauhan, S. Dube, A. Kapoor, K. Kothekar, A. Rane, S. Sharma, H. Behnamian, S. Chenarani, E. Eskandari Tadavani, S. M. Etesami, A. Fahim, M. Khakzad, M. Mohammadi Najafabadi, M. Naseri, S. Paktinat Mehdiabadi, F. Rezaei Hosseinabadi, B. Safarzadeh, M. Zeinali, M. Felcini, M. Grunewald, M. Abbrescia, C. Calabria, C. Caputo, A. Colaleo, D. Creanza, L. Cristella, N. De Filippis, M. De Palma, L. Fiore, G. Iaselli, G. Maggi, M. Maggi, G. Miniello, S. My, S. Nuzzo, A. Pompili, G. Pugliese, R. Radogna, A. Ranieri, G. Selvaggi, L. Silvestris, R. Venditti, P. Verwilligen, G. Abbiendi, C. Battilana, D. Bonacorsi, S. Braibant-Giacomelli, L. Brigliadori, R. Campanini, P. Capiluppi, A. Castro, F. R. Cavallo, S. S. Chhibra, G. Codispoti, M. Cuffiani, G. M. Dallavalle, F. Fabbri, A. Fanfani, D. Fasanella, P. Giacomelli, C. Grandi, L. Guiducci, S. Marcellini, G. Masetti, A. Montanari, F. L. Navarria, A. Perrotta, A. M. Rossi, T. Rovelli, G. P. Siroli, N. Tosi, S. Albergo, M. Chiorboli, S. Costa, A. Di Mattia, F. Giordano, R. Potenza, A. Tricomi, C. Tuve, G. Barbagli, V. Ciulli, C. Civinini, R. D’Alessandro, E. Focardi, V. Gori, P. Lenzi, M. Meschini, S. Paoletti, G. Sguazzoni, L. Viliani, L. Benussi, S. Bianco, F. Fabbri, D. Piccolo, F. Primavera, V. Calvelli, F. Ferro, M. Lo Vetere, M. R. Monge, E. Robutti, S. Tosi, L. Brianza, M. E. Dinardo, S. Fiorendi, S. Gennai, A. Ghezzi, P. Govoni, S. Malvezzi, R. A. Manzoni, B. Marzocchi, D. Menasce, L. Moroni, M. Paganoni, D. Pedrini, S. Pigazzini, S. Ragazzi, T. Tabarelli de Fatis, S. Buontempo, N. Cavallo, G. De Nardo, S. Di Guida, M. Esposito, F. Fabozzi, A. O. M. Iorio, G. Lanza, L. Lista, S. Meola, P. Paolucci, C. Sciacca, F. Thyssen, P. Azzi, N. Bacchetta, L. Benato, D. Bisello, A. Boletti, R. Carlin, A. Carvalho Antunes De Oliveira, P. Checchia, M. Dall’Osso, P. De Castro Manzano, T. Dorigo, U. Dosselli, F. Gasparini, U. Gasparini, A. Gozzelino, S. Lacaprara, M. Margoni, A. T. Meneguzzo, J. Pazzini, N. Pozzobon, P. Ronchese, F. Simonetto, E. Torassa, M. Zanetti, P. Zotto, A. Zucchetta, G. Zumerle, A. Braghieri, A. Magnani, P. Montagna, S. P. Ratti, V. Re, C. Riccardi, P. Salvini, I. Vai, P. Vitulo, L. Alunni Solestizi, G. M. Bilei, D. Ciangottini, L. Fanò, P. Lariccia, R. Leonardi, G. Mantovani, M. Menichelli, A. Saha, A. Santocchia, K. Androsov, P. Azzurri, G. Bagliesi, J. Bernardini, T. Boccali, R. Castaldi, M. A. Ciocci, R. Dell’Orso, S. Donato, G. Fedi, A. Giassi, M. T. Grippo, F. Ligabue, T. Lomtadze, L. Martini, A. Messineo, F. Palla, A. Rizzi, A. Savoy-Navarro, P. Spagnolo, R. Tenchini, G. Tonelli, A. Venturi, P. G. Verdini, L. Barone, F. Cavallari, M. Cipriani, G. D’imperio, D. Del Re, M. Diemoz, S. Gelli, C. Jorda, E. Longo, F. Margaroli, P. Meridiani, G. Organtini, R. Paramatti, F. Preiato, S. Rahatlou, C. Rovelli, F. Santanastasio, N. Amapane, R. Arcidiacono, S. Argiro, M. Arneodo, N. Bartosik, R. Bellan, C. Biino, N. Cartiglia, F. Cenna, M. Costa, R. Covarelli, A. Degano, N. Demaria, L. Finco, B. Kiani, C. Mariotti, S. Maselli, E. Migliore, V. Monaco, E. Monteil, M. M. Obertino, L. Pacher, N. Pastrone, M. Pelliccioni, G. L. Pinna Angioni, F. Ravera, A. Romero, M. Ruspa, R. Sacchi, K. Shchelina, V. Sola, A. Solano, A. Staiano, P. Traczyk, S. Belforte, M. Casarsa, F. Cossutti, G. Della Ricca, C. La Licata, A. Schizzi, A. Zanetti, D. H. Kim, G. N. Kim, M. S. Kim, S. Lee, S. W. Lee, Y. D. Oh, S. Sekmen, D. C. Son, Y. C. Yang, A. Lee, J. A. Brochero Cifuentes, T. J. Kim, S. Cho, S. Choi, Y. Go, D. Gyun, S. Ha, B. Hong, Y. Jo, Y. Kim, B. Lee, K. Lee, K. S. Lee, S. Lee, J. Lim, S. K. Park, Y. Roh, J. Almond, J. Kim, S. B. Oh, S. H. Seo, U. K. Yang, H. D. Yoo, G. B. Yu, M. Choi, H. Kim, H. Kim, J. H. Kim, J. S. H. Lee, I. C. Park, G. Ryu, M. S. Ryu, Y. Choi, J. Goh, C. Hwang, J. Lee, I. Yu, V. Dudenas, A. Juodagalvis, J. Vaitkus, I. Ahmed, Z. A. Ibrahim, J. R. Komaragiri, M. A. B. Md Ali, F. Mohamad Idris, W. A. T. Wan Abdullah, M. N. Yusli, Z. Zolkapli, H. Castilla-Valdez, E. De La Cruz-Burelo, I. Heredia-De La Cruz, A. Hernandez-Almada, R. Lopez-Fernandez, J. Mejia Guisao, A. Sanchez-Hernandez, S. Carrillo Moreno, C. Oropeza Barrera, F. Vazquez Valencia, S. Carpinteyro, I. Pedraza, H. A. Salazar Ibarguen, C. Uribe Estrada, A. Morelos Pineda, D. Krofcheck, P. H. Butler, A. Ahmad, M. Ahmad, Q. Hassan, H. R. Hoorani, W. A. Khan, M A. Shah, M. Shoaib, M. Waqas, H. Bialkowska, M. Bluj, B. Boimska, T. Frueboes, M. Górski, M. Kazana, K. Nawrocki, K. Romanowska-Rybinska, M. Szleper, P. Zalewski, K. Bunkowski, A. Byszuk, K. Doroba, A. Kalinowski, M. Konecki, J. Krolikowski, M. Misiura, M. Olszewski, M. Walczak, P. Bargassa, C. Beirão Da Cruz E Silva, A. Di Francesco, P. Faccioli, P. G. Ferreira Parracho, M. Gallinaro, J. Hollar, N. Leonardo, L. Lloret Iglesias, M. V. Nemallapudi, J. Rodrigues Antunes, J. Seixas, O. Toldaiev, D. Vadruccio, J. Varela, P. Vischia, S. Afanasiev, P. Bunin, M. Gavrilenko, I. Golutvin, I. Gorbunov, A. Kamenev, V. Karjavin, A. Lanev, A. Malakhov, V. Matveev, P. Moisenz, V. Palichik, V. Perelygin, S. Shmatov, S. Shulha, N. Skatchkov, V. Smirnov, N. Voytishin, A. Zarubin, L. Chtchipounov, V. Golovtsov, Y. Ivanov, V. Kim, E. Kuznetsova, V. Murzin, V. Oreshkin, V. Sulimov, A. Vorobyev, Yu. Andreev, A. Dermenev, S. Gninenko, N. Golubev, A. Karneyeu, M. Kirsanov, N. Krasnikov, A. Pashenkov, D. Tlisov, A. Toropin, V. Epshteyn, V. Gavrilov, N. Lychkovskaya, V. Popov, l. Pozdnyakov, G. Safronov, A. Spiridonov, M. Toms, E. Vlasov, A. Zhokin, M. Chadeeva, M. Danilov, O. Markin, V. Andreev, M. Azarkin, I. Dremin, M. Kirakosyan, A. Leonidov, S. V. Rusakov, A. Terkulov, A. Baskakov, A. Belyaev, E. Boos, M. Dubinin, L. Dudko, A. Ershov, A. Gribushin, V. Klyukhin, O. Kodolova, I. Lokhtin, I. Miagkov, S. Obraztsov, S. Petrushanko, V. Savrin, A Snigirev, I. Azhgirey, I. Bayshev, S. Bitioukov, D. Elumakhov, V. Kachanov, A. Kalinin, D. Konstantinov, V. Krychkine, V. Petrov, R. Ryutin, A. Sobol, S. Troshin, N. Tyurin, A. Uzunian, A. Volkov, P. Adzic, P. Cirkovic, D. Devetak, J. Milosevic, V. Rekovic, J. Alcaraz Maestre, E. Calvo, M. Cerrada, M. Chamizo Llatas, N. Colino, B. De La Cruz, A. Delgado Peris, A. Escalante Del Valle, C. Fernandez Bedoya, J. P. Fernández Ramos, J. Flix, M. C. Fouz, P. Garcia-Abia, O. Gonzalez Lopez, S. Goy Lopez, J. M. Hernandez, M. I. Josa, E. Navarro De Martino, A. Pérez-Calero Yzquierdo, J. Puerta Pelayo, A. Quintario Olmeda, I. Redondo, L. Romero, M. S. Soares, J. F. de Trocóniz, M. Missiroli, D. Moran, J. Cuevas, J. Fernandez Menendez, I. Gonzalez Caballero, J. R. González Fernández, E. Palencia Cortezon, S. Sanchez Cruz, I. Suárez Andrés, J. M. Vizan Garcia, I. J. Cabrillo, A. Calderon, J. R. Castiñeiras De Saa, E. Curras, M. Fernandez, J. Garcia-Ferrero, G. Gomez, A. Lopez Virto, J. Marco, C. Martinez Rivero, F. Matorras, J. Piedra Gomez, T. Rodrigo, A. Ruiz-Jimeno, L. Scodellaro, N. Trevisani, I. Vila, R. Vilar Cortabitarte, D. Abbaneo, E. Auffray, G. Auzinger, M. Bachtis, P. Baillon, A. H. Ball, D. Barney, P. Bloch, A. Bocci, A. Bonato, C. Botta, T. Camporesi, R. Castello, M. Cepeda, G. Cerminara, M. D’Alfonso, D. d’Enterria, A. Dabrowski, V. Daponte, A. David, M. De Gruttola, F. De Guio, A. De Roeck, E. Di Marco, M. Dobson, M. Dordevic, B. Dorney, T. du Pree, D. Duggan, M. Dünser, N. Dupont, A. Elliott-Peisert, S. Fartoukh, G. Franzoni, J. Fulcher, W. Funk, D. Gigi, K. Gill, M. Girone, F. Glege, D. Gulhan, S. Gundacker, M. Guthoff, J. Hammer, P. Harris, J. Hegeman, V. Innocente, P. Janot, H. Kirschenmann, V. Knünz, A. Kornmayer, M. J. Kortelainen, K. Kousouris, M. Krammer, P. Lecoq, C. Lourenço, M. T. Lucchini, L. Malgeri, M. Mannelli, A. Martelli, F. Meijers, S. Mersi, E. Meschi, F. Moortgat, S. Morovic, M. Mulders, H. Neugebauer, S. Orfanelli, L. Orsini, L. Pape, E. Perez, M. Peruzzi, A. Petrilli, G. Petrucciani, A. Pfeiffer, M. Pierini, A. Racz, T. Reis, G. Rolandi, M. Rovere, M. Ruan, H. Sakulin, J. B. Sauvan, C. Schäfer, C. Schwick, M. Seidel, A. Sharma, P. Silva, M. Simon, P. Sphicas, J. Steggemann, M. Stoye, Y. Takahashi, M. Tosi, D. Treille, A. Triossi, A. Tsirou, V. Veckalns, G. I. Veres, N. Wardle, A. Zagozdzinska, W. D. Zeuner, W. Bertl, K. Deiters, W. Erdmann, R. Horisberger, Q. Ingram, H. C. Kaestli, D. Kotlinski, U. Langenegger, T. Rohe, F. Bachmair, L. Bäni, L. Bianchini, B. Casal, G. Dissertori, M. Dittmar, M. Donegà, P. Eller, C. Grab, C. Heidegger, D. Hits, J. Hoss, G. Kasieczka, P. Lecomte, W. Lustermann, B. Mangano, M. Marionneau, P. Martinez Ruiz del Arbol, M. Masciovecchio, M. T. Meinhard, D. Meister, F. Micheli, P. Musella, F. Nessi-Tedaldi, F. Pandolfi, J. Pata, F. Pauss, G. Perrin, L. Perrozzi, M. Quittnat, M. Rossini, M. Schönenberger, A. Starodumov, M. Takahashi, V. R. Tavolaro, K. Theofilatos, R. Wallny, T. K. Aarrestad, C. Amsler, L. Caminada, M. F. Canelli, V. Chiochia, A. De Cosa, C. Galloni, A. Hinzmann, T. Hreus, B. Kilminster, C. Lange, J. Ngadiuba, D. Pinna, G. Rauco, P. Robmann, D. Salerno, Y. Yang, V. Candelise, T. H. Doan, Sh. Jain, R. Khurana, M. Konyushikhin, C. M. Kuo, W. Lin, Y. J. Lu, A. Pozdnyakov, S. S. Yu, Arun Kumar, P. Chang, Y. H. Chang, Y. W. Chang, Y. Chao, K. F. Chen, P. H. Chen, C. Dietz, F. Fiori, W.-S. Hou, Y. Hsiung, Y. F. Liu, R.-S. Lu, M. Miñano Moya, E. Paganis, A. Psallidas, J. F. Tsai, Y. M. Tzeng, B. Asavapibhop, G. Singh, N. Srimanobhas, N. Suwonjandee, A. Adiguzel, S. Cerci, S. Damarseckin, Z. S. Demiroglu, C. Dozen, I. Dumanoglu, S. Girgis, G. Gokbulut, Y. Guler, E. Gurpinar, I. Hos, E. E. Kangal, O. Kara, A. Kayis Topaksu, U. Kiminsu, M. Oglakci, G. Onengut, K. Ozdemir, D. Sunar Cerci, B. Tali, S. Turkcapar, I. S. Zorbakir, C. Zorbilmez, B. Bilin, S. Bilmis, B. Isildak, G. Karapinar, M. Yalvac, M. Zeyrek, E. Gülmez, M. Kaya, O. Kaya, E. A. Yetkin, T. Yetkin, A. Cakir, K. Cankocak, S. Sen, B. Grynyov, L. Levchuk, P. Sorokin, R. Aggleton, F. Ball, L. Beck, J. J. Brooke, D. Burns, E. Clement, D. Cussans, H. Flacher, J. Goldstein, M. Grimes, G. P. Heath, H. F. Heath, J. Jacob, L. Kreczko, C. Lucas, D. M. Newbold, S. Paramesvaran, A. Poll, T. Sakuma, S. Seif El Nasr-Storey, D. Smith, V. J. Smith, K. W. Bell, A. Belyaev, C. Brew, R. M. Brown, L. Calligaris, D. Cieri, D. J. A. Cockerill, J. A. Coughlan, K. Harder, S. Harper, E. Olaiya, D. Petyt, C. H. Shepherd-Themistocleous, A. Thea, I. R. Tomalin, T. Williams, M. Baber, R. Bainbridge, O. Buchmuller, A. Bundock, D. Burton, S. Casasso, M. Citron, D. Colling, L. Corpe, P. Dauncey, G. Davies, A. De Wit, M. Della Negra, P. Dunne, A. Elwood, D. Futyan, Y. Haddad, G. Hall, G. Iles, R. Lane, C. Laner, R. Lucas, L. Lyons, A.-M. Magnan, S. Malik, L. Mastrolorenzo, J. Nash, A. Nikitenko, J. Pela, B. Penning, M. Pesaresi, D. M. Raymond, A. Richards, A. Rose, C. Seez, A. Tapper, K. Uchida, M. Vazquez Acosta, T. Virdee, S. C. Zenz, J. E. Cole, P. R. Hobson, A. Khan, P. Kyberd, D. Leslie, I. D. Reid, P. Symonds, L. Teodorescu, M. Turner, A. Borzou, K. Call, J. Dittmann, K. Hatakeyama, H. Liu, N. Pastika, O. Charaf, S. I. Cooper, C. Henderson, P. Rumerio, D. Arcaro, A. Avetisyan, T. Bose, D. Gastler, D. Rankin, C. Richardson, J. Rohlf, L. Sulak, D. Zou, G. Benelli, E. Berry, D. Cutts, A. Garabedian, J. Hakala, U. Heintz, J. M. Hogan, O. Jesus, E. Laird, G. Landsberg, Z. Mao, M. Narain, S. Piperov, S. Sagir, E. Spencer, R. Syarif, R. Breedon, G. Breto, D. Burns, M. Calderon De La Barca Sanchez, S. Chauhan, M. Chertok, J. Conway, R. Conway, P. T. Cox, R. Erbacher, C. Flores, G. Funk, M. Gardner, W. Ko, R. Lander, C. Mclean, M. Mulhearn, D. Pellett, J. Pilot, F. Ricci-Tam, S. Shalhout, J. Smith, M. Squires, D. Stolp, M. Tripathi, S. Wilbur, R. Yohay, R. Cousins, P. Everaerts, A. Florent, J. Hauser, M. Ignatenko, D. Saltzberg, E. Takasugi, V. Valuev, M. Weber, K. Burt, R. Clare, J. Ellison, J. W. Gary, G. Hanson, J. Heilman, P. Jandir, E. Kennedy, F. Lacroix, O. R. Long, M. Malberti, M. Olmedo Negrete, M. I. Paneva, A. Shrinivas, H. Wei, S. Wimpenny, B. R. Yates, J. G. Branson, G. B. Cerati, S. Cittolin, M. Derdzinski, R. Gerosa, A. Holzner, D. Klein, V. Krutelyov, J. Letts, I. Macneill, D. Olivito, S. Padhi, M. Pieri, M. Sani, V. Sharma, S. Simon, M. Tadel, A. Vartak, S. Wasserbaech, C. Welke, J. Wood, F. Würthwein, A. Yagil, G. Zevi Della Porta, N. Amin, R. Bhandari, J. Bradmiller-Feld, C. Campagnari, A. Dishaw, V. Dutta, K. Flowers, M. Franco Sevilla, P. Geffert, C. George, F. Golf, L. Gouskos, J. Gran, R. Heller, J. Incandela, N. Mccoll, S. D. Mullin, A. Ovcharova, J. Richman, D. Stuart, I. Suarez, C. West, J. Yoo, D. Anderson, A. Apresyan, J. Bendavid, A. Bornheim, J. Bunn, Y. Chen, J. Duarte, A. Mott, H. B. Newman, C. Pena, M. Spiropulu, J. R. Vlimant, S. Xie, R. Y. Zhu, M. B. Andrews, V. Azzolini, B. Carlson, T. Ferguson, M. Paulini, J. Russ, M. Sun, H. Vogel, I. Vorobiev, J. P. Cumalat, W. T. Ford, F. Jensen, A. Johnson, M. Krohn, T. Mulholland, K. Stenson, S. R. Wagner, J. Alexander, J. Chaves, J. Chu, S. Dittmer, K. Mcdermott, N. Mirman, G. Nicolas Kaufman, J. R. Patterson, A. Rinkevicius, A. Ryd, L. Skinnari, L. Soffi, S. M. Tan, Z. Tao, J. Thom, J. Tucker, P. Wittich, M. Zientek, D. Winn, S. Abdullin, M. Albrow, G. Apollinari, S. Banerjee, L. A. T. Bauerdick, A. Beretvas, J. Berryhill, P. C. Bhat, G. Bolla, K. Burkett, J. N. Butler, H. W. K. Cheung, F. Chlebana, S. Cihangir, M. Cremonesi, V. D. Elvira, I. Fisk, J. Freeman, E. Gottschalk, L. Gray, D. Green, S. Grünendahl, O. Gutsche, D. Hare, R. M. Harris, S. Hasegawa, J. Hirschauer, Z. Hu, B. Jayatilaka, S. Jindariani, M. Johnson, U. Joshi, B. Klima, B. Kreis, S. Lammel, J. Linacre, D. Lincoln, R. Lipton, T. Liu, R. Lopes De Sá, J. Lykken, K. Maeshima, N. Magini, J. M. Marraffino, S. Maruyama, D. Mason, P. McBride, P. Merkel, S. Mrenna, S. Nahn, C. Newman-Holmes, V. O’Dell, K. Pedro, O. Prokofyev, G. Rakness, L. Ristori, E. Sexton-Kennedy, A. Soha, W. J. Spalding, L. Spiegel, S. Stoynev, N. Strobbe, L. Taylor, S. Tkaczyk, N. V. Tran, L. Uplegger, E. W. Vaandering, C. Vernieri, M. Verzocchi, R. Vidal, M. Wang, H. A. Weber, A. Whitbeck, D. Acosta, P. Avery, P. Bortignon, D. Bourilkov, A. Brinkerhoff, A. Carnes, M. Carver, D. Curry, S. Das, R. D. Field, I. K. Furic, J. Konigsberg, A. Korytov, P. Ma, K. Matchev, H. Mei, P. Milenovic, G. Mitselmakher, D. Rank, L. Shchutska, D. Sperka, L. Thomas, J. Wang, S. Wang, J. Yelton, S. Linn, P. Markowitz, G. Martinez, J. L. Rodriguez, A. Ackert, J. R. Adams, T. Adams, A. Askew, S. Bein, B. Diamond, S. Hagopian, V. Hagopian, K. F. Johnson, A. Khatiwada, H. Prosper, A. Santra, M. Weinberg, M. M. Baarmand, V. Bhopatkar, S. Colafranceschi, M. Hohlmann, D. Noonan, T. Roy, F. Yumiceva, M. R. Adams, L. Apanasevich, D. Berry, R. R. Betts, I. Bucinskaite, R. Cavanaugh, O. Evdokimov, L. Gauthier, C. E. Gerber, D. J. Hofman, P. Kurt, C. O’Brien, l. D. Sandoval Gonzalez, P. Turner, N. Varelas, H. Wang, Z. Wu, M. Zakaria, J. Zhang, B. Bilki, W. Clarida, K. Dilsiz, S. Durgut, R. P. Gandrajula, M. Haytmyradov, V. Khristenko, J.-P. Merlo, H. Mermerkaya, A. Mestvirishvili, A. Moeller, J. Nachtman, H. Ogul, Y. Onel, F. Ozok, A. Penzo, C. Snyder, E. Tiras, J. Wetzel, K. Yi, I. Anderson, B. Blumenfeld, A. Cocoros, N. Eminizer, D. Fehling, L. Feng, A. V. Gritsan, P. Maksimovic, M. Osherson, J. Roskes, U. Sarica, M. Swartz, M. Xiao, Y. Xin, C. You, A. Al-bataineh, P. Baringer, A. Bean, J. Bowen, C. Bruner, J. Castle, R. P. Kenny, A. Kropivnitskaya, D. Majumder, W. Mcbrayer, M. Murray, S. Sanders, R. Stringer, J. D. Tapia Takaki, Q. Wang, A. Ivanov, K. Kaadze, S. Khalil, M. Makouski, Y. Maravin, A. Mohammadi, L. K. Saini, N. Skhirtladze, S. Toda, D. Lange, F. Rebassoo, D. Wright, C. Anelli, A. Baden, O. Baron, A. Belloni, B. Calvert, S. C. Eno, C. Ferraioli, J. A. Gomez, N. J. Hadley, S. Jabeen, R. G. Kellogg, T. Kolberg, J. Kunkle, Y. Lu, A. C. Mignerey, Y. H. Shin, A. Skuja, M. B. Tonjes, S. C. Tonwar, D. Abercrombie, B. Allen, A. Apyan, R. Barbieri, A. Baty, R. Bi, K. Bierwagen, S. Brandt, W. Busza, I. A. Cali, Z. Demiragli, L. Di Matteo, G. Gomez Ceballos, M. Goncharov, D. Hsu, Y. Iiyama, G. M. Innocenti, M. Klute, D. Kovalskyi, K. Krajczar, Y. S. Lai, Y.-J. Lee, A. Levin, P. D. Luckey, A. C. Marini, C. Mcginn, C. Mironov, S. Narayanan, X. Niu, C. Paus, C. Roland, G. Roland, J. Salfeld-Nebgen, G. S. F. Stephans, K. Sumorok, K. Tatar, M. Varma, D. Velicanu, J. Veverka, J. Wang, T. W. Wang, B. Wyslouch, M. Yang, V. Zhukova, A. C. Benvenuti, R. M. Chatterjee, A. Evans, A. Finkel, A. Gude, P. Hansen, S. Kalafut, S. C. Kao, Y. Kubota, Z. Lesko, J. Mans, S. Nourbakhsh, N. Ruckstuhl, R. Rusack, N. Tambe, J. Turkewitz, J. G. Acosta, S. Oliveros, E. Avdeeva, R. Bartek, K. Bloom, S. Bose, D. R. Claes, A. Dominguez, C. Fangmeier, R. Gonzalez Suarez, R. Kamalieddin, D. Knowlton, I. Kravchenko, A. Malta Rodrigues, F. Meier, J. Monroy, J. E. Siado, G. R. Snow, B. Stieger, M. Alyari, J. Dolen, J. George, A. Godshalk, C. Harrington, I. Iashvili, J. Kaisen, A. Kharchilava, A. Kumar, A. Parker, S. Rappoccio, B. Roozbahani, G. Alverson, E. Barberis, D. Baumgartel, A. Hortiangtham, A. Massironi, D. M. Morse, D. Nash, T. Orimoto, R. Teixeira De Lima, D. Trocino, R.-J. Wang, D. Wood, S. Bhattacharya, K. A. Hahn, A. Kubik, J. F. Low, N. Mucia, N. Odell, B. Pollack, M. H. Schmitt, K. Sung, M. Trovato, M. Velasco, N. Dev, M. Hildreth, K. Hurtado Anampa, C. Jessop, D. J. Karmgard, N. Kellams, K. Lannon, N. Marinelli, F. Meng, C. Mueller, Y. Musienko, M. Planer, A. Reinsvold, R. Ruchti, G. Smith, S. Taroni, N. Valls, M. Wayne, M. Wolf, A. Woodard, J. Alimena, L. Antonelli, J. Brinson, B. Bylsma, L. S. Durkin, S. Flowers, B. Francis, A. Hart, C. Hill, R. Hughes, W. Ji, B. Liu, W. Luo, D. Puigh, B. L. Winer, H. W. Wulsin, S. Cooperstein, O. Driga, P. Elmer, J. Hardenbrook, P. Hebda, J. Luo, D. Marlow, T. Medvedeva, M. Mooney, J. Olsen, C. Palmer, P. Piroué, D. Stickland, C. Tully, A. Zuranski, S. Malik, A. Barker, V. E. Barnes, D. Benedetti, S. Folgueras, L. Gutay, M. K. Jha, M. Jones, A. W. Jung, K. Jung, D. H. Miller, N. Neumeister, B. C. Radburn-Smith, X. Shi, J. Sun, A. Svyatkovskiy, F. Wang, W. Xie, L. Xu, N. Parashar, J. Stupak, A. Adair, B. Akgun, Z. Chen, K. M. Ecklund, F. J. M. Geurts, M. Guilbaud, W. Li, B. Michlin, M. Northup, B. P. Padley, R. Redjimi, J. Roberts, J. Rorie, Z. Tu, J. Zabel, B. Betchart, A. Bodek, P. de Barbaro, R. Demina, t. Duh, Y. t. Ferbel, M. Galanti, A. Garcia-Bellido, J. Han, O. Hindrichs, A. Khukhunaishvili, K. H. Lo, P. Tan, M. Verzetti, J. P. Chou, E. Contreras-Campana, Y. Gershtein, T. A. Gómez Espinosa, E. Halkiadakis, M. Heindl, D. Hidas, E. Hughes, S. Kaplan, R. Kunnawalkam Elayavalli, S. Kyriacou, A. Lath, K. Nash, H. Saka, S. Salur, S. Schnetzer, D. Sheffield, S. Somalwar, R. Stone, S. Thomas, P. Thomassen, M. Walker, M. Foerster, J. Heideman, G. Riley, K. Rose, S. Spanier, K. Thapa, O. Bouhali, A. Celik, M. Dalchenko, M. De Mattia, A. Delgado, S. Dildick, R. Eusebi, J. Gilmore, T. Huang, E. Juska, T. Kamon, R. Mueller, Y. Pakhotin, R. Patel, A. Perloff, L. Perniè, D. Rathjens, A. Rose, A. Safonov, A. Tatarinov, K. A. Ulmer, N. Akchurin, C. Cowden, J. Damgov, C. Dragoiu, P. R. Dudero, J. Faulkner, S. Kunori, K. Lamichhane, S. W. Lee, T. Libeiro, S. Undleeb, I. Volobouev, Z. Wang, A. G. Delannoy, S. Greene, A. Gurrola, R. Janjam, W. Johns, C. Maguire, A. Melo, H. Ni, P. Sheldon, S. Tuo, J. Velkovska, Q. Xu, M. W. Arenton, P. Barria, B. Cox, J. Goodell, R. Hirosky, A. Ledovskoy, H. Li, C. Neu, T. Sinthuprasith, X. Sun, Y. Wang, E. Wolfe, F. Xia, C. Clarke, R. Harr, P. E. Karchin, P. Lamichhane, J. Sturdy, D. A. Belknap, S. Dasu, L. Dodd, S. Duric, B. Gomber, M. Grothe, M. Herndon, A. Hervé, P. Klabbers, A. Lanaro, A. Levine, K. Long, R. Loveless, I. Ojalvo, T. Perry, G. A. Pierro, G. Polese, T. Ruggles, A. Savin, A. Sharma, N. Smith, W. H. Smith, D. Taylor, N. Woods, [Authorinst]The CMS Collaboration

**Affiliations:** 1 Yerevan Physics Institute, Yerevan, Armenia; 2 Institut für Hochenergiephysik der OeAW, Vienna, Austria; 3 National Centre for Particle and High Energy Physics, Minsk, Belarus; 4 Universiteit Antwerpen, Antwerp, Belgium; 5 Vrije Universiteit Brussel, Brussels, Belgium; 6 Université Libre de Bruxelles, Brussels, Belgium; 7 Ghent University, Ghent, Belgium; 8 Université Catholique de Louvain, Louvain-la-Neuve, Belgium; 9 Université de Mons, Mons, Belgium; 10 Centro Brasileiro de Pesquisas Fisicas, Rio de Janeiro, Brazil; 11 Universidade do Estado do Rio de Janeiro, Rio de Janeiro, Brazil; 12 Universidade Estadual Paulista, Universidade Federal do ABC, São Paulo, Brazil; 13 Institute for Nuclear Research and Nuclear Energy, Sofia, Bulgaria; 14 University of Sofia, Sofia, Bulgaria; 15 Beihang University, Beijing, China; 16 Institute of High Energy Physics, Beijing, China; 17 State Key Laboratory of Nuclear Physics and Technology, Peking University, Beijing, China; 18 Universidad de Los Andes, Bogotá, Colombia; 19 Faculty of Electrical Engineering, Mechanical Engineering and Naval Architecture, University of Split, Split, Croatia; 20 Faculty of Science, University of Split, Split, Croatia; 21 Institute Rudjer Boskovic, Zagreb, Croatia; 22 University of Cyprus, Nicosia, Cyprus; 23 Charles University, Prague, Czech Republic; 24 Universidad San Francisco de Quito, Quito, Ecuador; 25 Academy of Scientific Research and Technology of the Arab Republic of Egypt, Egyptian Network of High Energy Physics, Cairo, Egypt; 26 National Institute of Chemical Physics and Biophysics, Tallinn, Estonia; 27 Department of Physics, University of Helsinki, Helsinki, Finland; 28 Helsinki Institute of Physics, Helsinki, Finland; 29 Lappeenranta University of Technology, Lappeenranta, Finland; 30 DSM/IRFU, CEA/Saclay, Gif-sur-Yvette, France; 31 Laboratoire Leprince-Ringuet, Ecole Polytechnique, IN2P3-CNRS, Palaiseau, France; 32 Institut Pluridisciplinaire Hubert Curien, Université de Strasbourg, Université de Haute Alsace Mulhouse, CNRS/IN2P3, Strasbourg, France; 33 Centre de Calcul de l’Institut National de Physique Nucleaire et de Physique des Particules, CNRS/IN2P3, Villeurbanne, France; 34 Institut de Physique Nucléaire de Lyon, Université de Lyon, Université Claude Bernard Lyon 1, CNRS-IN2P3, Villeurbanne, France; 35 Georgian Technical University, Tbilisi, Georgia; 36 Tbilisi State University, Tbilisi, Georgia; 37 I. Physikalisches Institut, RWTH Aachen University, Aachen, Germany; 38 III. Physikalisches Institut A, RWTH Aachen University, Aachen, Germany; 39 III. Physikalisches Institut B, RWTH Aachen University, Aachen, Germany; 40 Deutsches Elektronen-Synchrotron, Hamburg, Germany; 41 University of Hamburg, Hamburg, Germany; 42 Institut für Experimentelle Kernphysik, Karlsruhe, Germany; 43 Institute of Nuclear and Particle Physics (INPP), NCSR Demokritos, Aghia Paraskevi, Greece; 44 National and Kapodistrian University of Athens, Athens, Greece; 45 University of Ioánnina, Ioánnina, Greece; 46 MTA-ELTE Lendület CMS Particle and Nuclear Physics Group, Eötvös Loránd University, Budapest, Hungary; 47 Wigner Research Centre for Physics, Budapest, Hungary; 48 Institute of Nuclear Research ATOMKI, Debrecen, Hungary; 49 University of Debrecen, Debrecen, Hungary; 50 National Institute of Science Education and Research, Bhubaneswar, India; 51 Panjab University, Chandigarh, India; 52 University of Delhi, Delhi, India; 53 Saha Institute of Nuclear Physics, Kolkata, India; 54 Indian Institute of Technology Madras, Madras, India; 55 Bhabha Atomic Research Centre, Mumbai, India; 56 Tata Institute of Fundamental Research, Mumbai, India; 57 Tata Institute of Fundamental Research-A, Mumbai, India; 58 Tata Institute of Fundamental Research-B, Mumbai, India; 59 Indian Institute of Science Education and Research (IISER), Pune, India; 60 Institute for Research in Fundamental Sciences (IPM), Tehran, Iran; 61 University College Dublin, Dublin, Ireland; 62 INFN Sezione di Bari, Università di Bari, Politecnico di Bari, Bari, Italy; 63 INFN Sezione di Bologna, Università di Bologna, Bologna, Italy; 64 INFN Sezione di Catania, Università di Catania, Catania, Italy; 65 INFN Sezione di Firenze, Università di Firenze, Florence, Italy; 66 INFN Laboratori Nazionali di Frascati, Frascati, Italy; 67 INFN Sezione di Genova, Università di Genova, Genoa, Italy; 68 INFN Sezione di Milano-Bicocca, Università di Milano-Bicocca, Milan, Italy; 69 INFN Sezione di Napoli, Università di Napoli ‘Federico II’, Naples, Italy, Università della Basilicata, Potenza, Italy, Università G. Marconi, Rome, Italy; 70 INFN Sezione di Padova, Università di Padova, Padua, Italy, Università di Trento, Trento, Italy; 71 INFN Sezione di Pavia, Università di Pavia, Pavia, Italy; 72 INFN Sezione di Perugia, Università di Perugia, Perugia, Italy; 73 INFN Sezione di Pisa, Università di Pisa, Scuola Normale Superiore di Pisa, Pisa, Italy; 74 INFN Sezione di Roma, Università di Roma, Rome, Italy; 75 INFN Sezione di Torino, Università di Torino, Turin, Italy, Università del Piemonte Orientale, Novara, Italy; 76 INFN Sezione di Trieste, Università di Trieste, Trieste, Italy; 77 Kyungpook National University, Daegu, Korea; 78 Chonbuk National University, Jeonju, Korea; 79 Hanyang University, Seoul, Korea; 80 Korea University, Seoul, Korea; 81 Seoul National University, Seoul, Korea; 82 University of Seoul, Seoul, Korea; 83 Sungkyunkwan University, Suwon, Korea; 84 Vilnius University, Vilnius, Lithuania; 85 National Centre for Particle Physics, Universiti Malaya, Kuala Lumpur, Malaysia; 86 Centro de Investigacion y de Estudios Avanzados del IPN, Mexico City, Mexico; 87 Universidad Iberoamericana, Mexico City, Mexico; 88 Benemerita Universidad Autonoma de Puebla, Puebla, Mexico; 89 Universidad Autónoma de San Luis Potosí, San Luis Potosí, Mexico; 90 University of Auckland, Auckland, New Zealand; 91 University of Canterbury, Christchurch, New Zealand; 92 National Centre for Physics, Quaid-I-Azam University, Islamabad, Pakistan; 93 National Centre for Nuclear Research, Swierk, Poland; 94 Faculty of Physics, Institute of Experimental Physics, University of Warsaw, Warsaw, Poland; 95 Laboratório de Instrumentação e Física Experimental de Partículas, Lisbon, Portugal; 96 Joint Institute for Nuclear Research, Dubna, Russia; 97 Petersburg Nuclear Physics Institute, Gatchina, St. Petersburg, Russia; 98 Institute for Nuclear Research, Moscow, Russia; 99 Institute for Theoretical and Experimental Physics, Moscow, Russia; 100 National Research Nuclear University ‘Moscow Engineering Physics Institute’ (MEPhI), Moscow, Russia; 101 P. N. Lebedev Physical Institute, Moscow, Russia; 102 Skobeltsyn Institute of Nuclear Physics, Lomonosov Moscow State University, Moscow, Russia; 103 State Research Center of Russian Federation, Institute for High Energy Physics, Protvino, Russia; 104 Faculty of Physics and Vinca Institute of Nuclear Sciences, University of Belgrade, Belgrade, Serbia; 105 Centro de Investigaciones Energéticas Medioambientales y Tecnológicas (CIEMAT), Madrid, Spain; 106 Universidad Autónoma de Madrid, Madrid, Spain; 107 Universidad de Oviedo, Oviedo, Spain; 108 Instituto de Física de Cantabria (IFCA), CSIC-Universidad de Cantabria, Santander, Spain; 109 CERN, European Organization for Nuclear Research, Geneva, Switzerland; 110 Paul Scherrer Institut, Villigen, Switzerland; 111 Institute for Particle Physics, ETH Zurich, Zurich, Switzerland; 112 Universität Zürich, Zurich, Switzerland; 113 National Central University, Chung-Li, Taiwan; 114 National Taiwan University (NTU), Taipei, Taiwan; 115 Department of Physics, Faculty of Science, Chulalongkorn University, Bangkok, Thailand; 116 Cukurova University, Adana, Turkey; 117 Physics Department, Middle East Technical University, Ankara, Turkey; 118 Bogazici University, Istanbul, Turkey; 119 Istanbul Technical University, Istanbul, Turkey; 120 Institute for Scintillation Materials of National Academy of Science of Ukraine, Kharkov, Ukraine; 121 National Scientific Center, Kharkov Institute of Physics and Technology, Kharkov, Ukraine; 122 University of Bristol, Bristol, UK; 123 Rutherford Appleton Laboratory, Didcot, UK; 124 Imperial College, London, UK; 125 Brunel University, Uxbridge, UK; 126 Baylor University, Waco, USA; 127 The University of Alabama, Tuscaloosa, USA; 128 Boston University, Boston, USA; 129 Brown University, Providence, USA; 130 University of California, Davis, Davis, USA; 131 University of California, Los Angeles, USA; 132 University of California, Riverside, Riverside, USA; 133 University of California, San Diego, La Jolla, USA; 134 University of California, Santa Barbara, Santa Barbara, USA; 135 California Institute of Technology, Pasadena, USA; 136 Carnegie Mellon University, Pittsburgh, USA; 137 University of Colorado Boulder, Boulder, USA; 138 Cornell University, Ithaca, USA; 139 Fairfield University, Fairfield, USA; 140 Fermi National Accelerator Laboratory, Batavia, USA; 141 University of Florida, Gainesville, USA; 142 Florida International University, Miami, USA; 143 Florida State University, Tallahassee, USA; 144 Florida Institute of Technology, Melbourne, USA; 145 University of Illinois at Chicago (UIC), Chicago, USA; 146 The University of Iowa, Iowa City, USA; 147 Johns Hopkins University, Baltimore, USA; 148 The University of Kansas, Lawrence, USA; 149 Kansas State University, Manhattan, USA; 150 Lawrence Livermore National Laboratory, Livermore, USA; 151 University of Maryland, College Park, USA; 152 Massachusetts Institute of Technology, Cambridge, USA; 153 University of Minnesota, Minneapolis, USA; 154 University of Mississippi, Oxford, USA; 155 University of Nebraska-Lincoln, Lincoln, USA; 156 State University of New York at Buffalo, Buffalo, USA; 157 Northeastern University, Boston, USA; 158 Northwestern University, Evanston, USA; 159 University of Notre Dame, Notre Dame, USA; 160 The Ohio State University, Columbus, USA; 161 Princeton University, Princeton, USA; 162 University of Puerto Rico, Mayaguez, USA; 163 Purdue University, West Lafayette, USA; 164 Purdue University Calumet, Hammond, USA; 165 Rice University, Houston, USA; 166 University of Rochester, Rochester, USA; 167 Rutgers, The State University of New Jersey, Piscataway, USA; 168 University of Tennessee, Knoxville, USA; 169 Texas A&M University, College Station, USA; 170 Texas Tech University, Lubbock, USA; 171 Vanderbilt University, Nashville, USA; 172 University of Virginia, Charlottesville, USA; 173 Wayne State University, Detroit, USA; 174 University of Wisconsin-Madison, Madison, WI USA; 175 CERN, Geneva, Switzerland

## Abstract

A search for new physics is performed using events with two isolated same-sign leptons, two or more jets, and missing transverse momentum. The results are based on a sample of proton–proton collisions at a center-of-mass energy of 13$\,\text {TeV}$ recorded with the CMS detector at the LHC, corresponding to an integrated luminosity of 2.3 $\mathrm{fb}^{1}$. Multiple search regions are defined by classifying events in terms of missing transverse momentum, the scalar sum of jet transverse momenta, the transverse mass associated with a $\mathrm {W}$ boson candidate, the number of jets, the number of $\mathrm{b}$ quark jets, and the transverse momenta of the leptons in the event. The analysis is sensitive to a wide variety of possible signals beyond the standard model. No excess above the standard model background expectation is observed. Constraints are set on various supersymmetric models, with gluinos and bottom squarks excluded for masses up to 1300 and 680$\,\text {GeV}$, respectively, at the 95 % confidence level. Upper limits on the cross sections for the production of two top quark-antiquark pairs (119$\,\text {fb}$) and two same-sign top quarks (1.7$\,\text {pb}$) are also obtained. Selection efficiencies and model independent limits are provided to allow further interpretations of the results.

## Introduction

Searches for new physics in final states with two leptons that have same-sign (SS) charges provide a powerful probe for searches of new physics, both because standard model (SM) processes with this signature are few and have low cross sections, and because this signature is produced in a large number of important new-physics scenarios. Examples of the latter include the production of supersymmetric (SUSY) particles [1, 2], Majorana neutrinos [3], vector-like quarks [4], and SS top quark pairs [5, 6]. In the SUSY framework [7–15], the SS signature can arise through gluino pair production. For example, the Majorana nature of the gluino allows gluino pairs to decay via SS charginos, yielding two SS W bosons. Gluino pair production can also yield four W bosons, e.g., from the decay of four top quarks, which may result in the SS dilepton final state. Alternatively, cascade decays of pair-produced squarks can lead to the SS dilepton signature. Searches for new physics in the SS channel have been previously performed at the CERN LHC by the ATLAS [16–18] and CMS [19–23] Collaborations.

This paper describes a search for new physics in the final state with two or more leptons and including a SS pair ($\mu ^\pm \mu ^\pm$, $\mu ^\pm \mathrm{e}^\pm$, or $\mathrm{e}^\pm \mathrm{e}^\pm$, where $\mu$ is a muon and $\mathrm {e}$ an electron). The analysis is based on proton–proton (pp) collision data at $\sqrt{s} = 13$ $\,\text {TeV}$, corresponding to an integrated luminosity of 2.3$\,\text {fb}^{-1}$ collected with the CMS detector in 2015. The search strategy resembles that used in our analysis of 19.5 $\mathrm{fb}^{1}$ of data collected at $\sqrt{s}=8\,\text {TeV}$ [23], which excluded gluino masses in the four top quark signature up to about 1050$\,\text {GeV}$. We design an inclusive analysis sensitive to a wide range of new-physics processes produced via strong interactions and yielding undetected particles in the final state. The interpretations of the results consider *R*-parity conserving SUSY models [24], as well as cross section limits on the production of two top quark-antiquark ($\mathrm{t}\overline{\mathrm{t}}$) pairs and of two SS top quarks. We also provide model independent limits to allow further interpretations of the results. With respect to Ref. [23], the kinematic regions are redefined and improvements in the event selection are implemented, both of which increase the sensitivity to new-physics scenarios at $\sqrt{s}=13\,\text {TeV}$.

## The CMS detector

The central feature of the CMS apparatus is a superconducting solenoid of 6 m internal diameter, providing a magnetic field of 3.8 T. Within the field volume are several particle detection systems. Charged-particle trajectories are measured with silicon pixel and strip trackers, covering $0 \le \phi < 2\pi$ in azimuth and $|\eta | < 2.5$ in pseudorapidity, where $\eta \equiv -\ln [\tan (\theta /2)]$ and $\theta$ is the polar angle of the trajectory of the particle with respect to the counterclockwise beam direction. The transverse momentum, namely the component of the momentum *p* in the plane orthogonal to the beam, is defined as $p_{\mathrm {T}} = p \sin \theta$. Surrounding the silicon trackers, a lead tungstate crystal electromagnetic calorimeter and a brass and scintillator hadron calorimeter provide energy measurements of electrons, photons, and hadronic jets in the range $|\eta | < 3.0$. Muons are identified and measured within $|\eta | < 2.4$ by gas-ionization detectors embedded in the steel flux-return yoke of the solenoid. Forward calorimeters on each side of the interaction point encompass $3.0< |\eta | < 5.0$. The CMS trigger consists of a two-stage system. The first level of the CMS trigger system, composed of custom hardware processors, uses information from the calorimeters and muon detectors to select events in a fixed time interval of less than 4$\,\upmu \text {s}$. The high-level trigger (HLT) processor farm further decreases the event rate from around 100 kHz to less than 1 kHz, before data storage. A more detailed description of the CMS detector can be found in Ref. [25].

## Event selection and Monte Carlo simulation

Events are selected with two sets of HLT algorithms. The first requires two very loosely isolated leptons, one satisfying $p_{\mathrm {T}} > 17\,\text {GeV}$ and the other satisfying $p_{\mathrm {T}} > 8\,\text {GeV}$ for a muon and 12$\,\text {GeV}$ for an electron. The isolation is evaluated with respect to nearby tracks for a muon and to both tracks and calorimetric objects for an electron. The second set of triggers selects events with lowered $p_{\mathrm {T}}$ thresholds of 8$\,\text {GeV}$ and without a restriction on the isolation, but requiring a hadronic activity $H_{\mathrm {T}} ^\text {HLT}>300\,\text {GeV}$, where $H_{\mathrm {T}} ^\text {HLT}$ is the scalar $p_{\mathrm {T}}$ sum of all jets with $p_{\mathrm {T}} > 40\,\text {GeV}$ and $|\eta | < 3.0$ identified by the HLT. Typical trigger efficiencies for leptons satisfying the selection criteria described below are 94 % (98 %) per muon (electron), with 100 % efficiency for the $H_{\mathrm {T}} ^\text {HLT}$ requirement.

In the subsequent analysis, muon candidates are reconstructed by combining information from the silicon tracker and the muon spectrometer in a global fit [26]. A selection is performed using the quality of the geometrical matching between the tracker and muon system measurements. We select muons with well-determined charge by imposing an additional criterion: $\delta p_{\mathrm {T}} (\mu )/p_{\mathrm {T}} (\mu ) < 0.2$, where $\delta p_{\mathrm {T}} (\mu )$ is the uncertainty in the measurement of the muon $p_{\mathrm {T}}$ from the global fit.

Electron candidates are reconstructed by combining clusters of energy in the electromagnetic calorimeter with tracks in the silicon tracker [27]. The identification is performed using a Boosted Decision Tree multivariate discriminant [28] based on shower shape and track quality variables. The nominal selection criteria are designed to provide a maximum rejection of electron candidates from multijet production while maintaining approximately 90 % efficiency for electrons from the decay of $\mathrm {W}$ or $\mathrm{Z}$ bosons. A relaxed selection on the multivariate discriminant is used to define “loose” criteria for electron identification. To improve the accuracy of the electron charge reconstruction, we require the position of the calorimeter deposit, relative to the linear projection of the deposits in the pixel detector to the inner calorimeter surface, to be consistent with the charge determination from the full track fit. Electrons originating from photon conversions are suppressed by rejecting candidates that are either without energy deposits in the innermost layers of the tracking system, or that are associated with a displaced vertex compatible with a photon conversion.

Lepton candidates are required to be consistent with originating from the collision vertex for which the summed $p_{\mathrm {T}} ^2$ of the associated physics objects is the largest. The transverse (longitudinal) impact parameter of the leptons must not exceed 0.5 (1.0) mm with respect to this vertex, and they must fulfill the requirement $|d_{\mathrm {3D}}|/\sigma (d_{\mathrm {3D}}) < 4$, where $d_{\mathrm {3D}}$ is the three-dimensional impact parameter with respect to the vertex, and $\sigma (d_{\mathrm {3D}})$ is its uncertainty from the track fit.

The charged leptons produced in decays of heavy particles, such as $\mathrm {W}$ and $\mathrm{Z}$ bosons or SUSY particles (“prompt” leptons), are typically spatially isolated from the hadronic activity in the event, while leptons produced in hadron decays or in photon conversions, as well as hadrons misidentified as leptons, are usually embedded in jets (“nonprompt” leptons). This distinction becomes less evident for systems with a high Lorentz boost, where decay products tend to overlap and jets may contribute to the energy deposition around prompt leptons. This problem is mitigated with an isolation definition constructed using the following three variables:

- the mini-isolation variable ($I_\text {mini}$) [29], computed as the ratio of the scalar $p_{\mathrm {T}}$ sum of charged hadrons, neutral hadrons, and photons within a cone of radius $\Delta R \equiv \sqrt{{(\Delta \eta )^2+(\Delta \phi )^2}}$ around the lepton candidate direction at the vertex, to the transverse momentum of the lepton candidate ($p_{\mathrm {T}} (\ell )$). The cone radius $\Delta R$ depends on $p_{\mathrm {T}} (\ell )$ as:
  1 $$\begin{aligned} \Delta R\left( p_{\mathrm {T}} (\ell )\right) = \dfrac{10\,\text {GeV}}{\min \left[ \max (p_{\mathrm {T}} (\ell ), 50\,\text {GeV}), 200\,\text {GeV} \right] }.\nonumber \\ \end{aligned}$$
  The varying isolation cone definition takes into account the increased collimation of the decay products of a hadron as its $p_{\mathrm {T}}$ increases, and it reduces the inefficiency from accidental overlap between the lepton and jets in a busy event environment. The momentum estimate of each particle is performed by the particle-flow (PF) algorithm [30, 31], which identifies individual particles through a combination of information from different detector components.
- the ratio of the $p_{\mathrm {T}}$ of the lepton to that of the closest jet within a distance $\Delta R = 0.4$:
  2 $$\begin{aligned} p_{\mathrm {T}} ^\text {ratio} = \dfrac{p_{\mathrm {T}} (\ell )}{p_{\mathrm {T}} (\text {jet})}, \end{aligned}$$
  where the definition of a jet is given below. In case of no jet within this distance, the value of $p_{\mathrm {T}} ^\text {ratio}$ is set to 1. The $p_{\mathrm {T}} ^\text {ratio}$ variable is a measure of the isolation in a larger cone and improves the performance of the isolation definition, especially for low-$p_{\mathrm {T}}$ nonprompt leptons, which are more likely than high-$p_{\mathrm {T}}$ leptons to appear in a jet that is wider than the $I_\text {mini}$ cone.
- the $p_{\mathrm {T}} ^\text {rel}$ variable [32], defined as the transverse momentum of the lepton relative to the residual momentum of the closest jet after lepton momentum subtraction:
  3 $$\begin{aligned} p_{\mathrm {T}} ^\text {rel} =\frac{\left| \left( {\vec {p}}(\text {jet})-{\vec {p}}(\ell )\right) \times {\vec {p}}(\ell )\right| }{|{\vec {p}}(\text {jet})-{\vec {p}}(\ell )|}. \end{aligned}$$
  This variable allows the identification of leptons that accidentally overlap with jets.

A lepton is considered to be isolated if the following condition is satisfied:

4 $$\begin{aligned} I_\text {mini} < I_1 \quad \text {AND}\quad (p_{\mathrm {T}} ^\text {ratio}> I_2 \ \text {OR} \ p_{\mathrm {T}} ^\text {rel} > I_3 ). \end{aligned}$$

The values of $I_{i}$, with $i = 1,2,3,$ depend on the lepton flavor: because the probability to misidentify a lepton is higher for electrons, tighter isolation values are used in this case (see Table 1). In addition, a “loose” isolation criterion is defined as $I_\text {mini} < 0.4$.

**Table 1:** Values of the isolation parameters used in Eq. (4)

Isolation variable	Muons	Electrons
$I_1$	0.16	0.12
$I_2$	0.76	0.80
$I_3\,(\text {GeV})$	7.2	7.2

Muons (electrons) are required to have $p_{\mathrm {T}} > 10$ (15) $\,\text {GeV}$ and $|\eta | < 2.4$ (2.5); at least one SS lepton pair with an invariant mass above $8\,\text {GeV}$ must be present in the event. In order to reduce backgrounds from inclusive production of the Z boson and from low-mass resonances decaying into lepton pairs, the SS pair is rejected if there is an additional lepton in the event that satisfies loose requirements and that forms an opposite-sign, same-flavor pair with an invariant mass less than 12$\,\text {GeV}$ or between 76 and 106$\,\text {GeV}$ with one of the two SS leptons.

Jets and missing transverse momentum ($E_{\mathrm {T}}^{\text {miss}}$) are reconstructed with the PF algorithm. We define $E_{\mathrm {T}}^{\text {miss}}$ as the magnitude of the vector sum of all PF candidate transverse momenta [33]. For jet clustering, the anti-$k_t$ algorithm [34] with a distance parameter of 0.4 is utilized. Jets are required to satisfy quality requirements [35] to remove those consistent with anomalous energy deposits. After the estimated contribution from additional $\mathrm {p}\mathrm {p}$ interactions in the same or adjacent bunch crossings (pileup) is subtracted, jet energies are corrected for residual nonuniformity and nonlinearity of the detector response using simulation and data. Jets are required to have $p_{\mathrm {T}} >40\,\text {GeV}$ and to lie within the tracker acceptance $|\eta |<2.4$. Jets must be separated from loosely identified leptons by $\Delta R > 0.4$, so that jets already employed for the calculation of lepton isolation variables are not considered further in the analysis. We require $N_\text {jet} \ge 2$, where $N_\text {jet}$ denotes the number of selected jets in the event. The hadronic activity in the event ($H_{\mathrm {T}}$) is defined as the scalar $p_{\mathrm {T}}$ sum of the selected jets.

To identify jets originating from b quarks, the combined secondary vertex algorithm CSVv2 [36] is used. Jets with $p_{\mathrm {T}} > 25\,\text {GeV}$ and $|\eta |<2.4$ are considered as b-tagged if they satisfy the requirements of the medium working point of the algorithm. These requirements result in approximately a 70 % efficiency for tagging a b quark jet, and a less than 1 % mistagging rate for light-quark and gluon jets in $\mathrm{t}\overline{\mathrm{t}}$ events. The number of b-tagged jets in the event is denoted as $N_{\mathrm{b}}$.

Monte Carlo (MC) simulation, which includes the contribution of pileup, is used to estimate the background from SM processes with prompt SS leptons (see Sect. 5) and to calculate the efficiency for various new-physics scenarios. The SM background samples are produced with the MadGraph5_aMC@NLO  2.2.2 generator [37] at leading order (LO) or next-to-leading order (NLO) accuracy in perturbative quantum chromodynamics, with the exception of diboson samples, which are produced with the powheg  v2 [38, 39] generator. The NNPDF3.0LO [40] parton distribution functions (PDFs) are used for the simulated samples generated at LO, and the NNPDF3.0NLO [40] PDFs for the samples generated at NLO. Parton showering and hadronization are described using the pythia  8.205 generator [41] with the CUETP8M1 tune [42, 43]. The CMS detector response for the background samples is modeled with the Geant4 package [44]. The signal samples are generated with MadGraph5_aMC@NLO at LO precision, including up to two additional partons in the matrix element calculations; parton showering and hadronization, as well as decays of SUSY particles, are simulated with pythia, while the detector simulation is performed with the CMS fast simulation package [45].

**Fig. 1:**
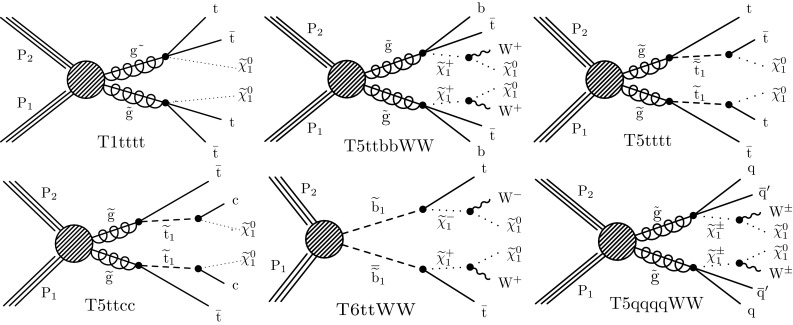
Diagrams illustrating the simplified SUSY models used in this analysis

## Search strategy

This analysis is designed as an inclusive search, sensitive to models matching two assumptions: a strong-interaction production mechanism, leading to relatively large hadronic activity, and the presence of undetected particles in the final state, yielding sizable $E_{\mathrm {T}}^{\text {miss}}$. In the process of defining the search strategy, *R*-parity conserving SUSY is taken as a guideline because of its rich variety of signatures. In this context, signal models that can lead to the experimental signature of SS lepton pairs differentiate themselves in the numbers of $\mathrm {W}$ bosons, b jets, and light-flavor jets produced in the decays of SUSY particles. In addition, the mass differences between the SUSY particles involved in the decay chains affect the energy spectra of the decay products, resulting in differences between the models in the distributions of kinematic quantities such as the $p_{\mathrm {T}}$ of the leptons, $H_{\mathrm {T}}$, and $E_{\mathrm {T}}^{\text {miss}}$.

We consider SUSY scenarios in the context of simplified models of new-particle production [46, 47]. Models with four $\mathrm {W}$ bosons and four b jets involve gluino pair production, followed by the decay of each gluino through a chain containing third-generation squarks. If the gluino is lighter than all squarks, and the top squark is the lightest squark, the gluino undergoes a three-body decay mediated by an off-shell top squark. If the dominant top squark decay is ${\widetilde{\mathrm{t}} _1} \rightarrow \mathrm{t}\widetilde{\chi }^{0}_{1}$, where $\widetilde{\chi }^{0}_{1}$ is the lightest neutralino, taken to be the stable, undetected, lightest SUSY particle (LSP), then the gluino three-body decay is $\widetilde{\mathrm{g}} \rightarrow \mathrm{t}\overline{\mathrm{t}} \widetilde{\chi }^{0}_{1}$ ($\mathrm {T}1\mathrm {tttt}$ model in Fig. 1, upper left). If instead the dominant top squark decay is ${\widetilde{\mathrm{t}} _1} \rightarrow \mathrm{b} \widetilde{\chi }^{+}_{1}$, the gluino three-body decay is $\widetilde{\mathrm{g}} \rightarrow \overline{\mathrm{t}}\mathrm{b} \widetilde{\chi }^{+}_{1}$ ($\mathrm {T}5\mathrm {ttbb}\mathrm {W}\mathrm {W}$ model in Fig. 1, upper middle); the latter signature can also arise if the bottom squark is the lightest squark and decays as ${\widetilde{\mathrm{b}} _1} \rightarrow \mathrm{t}\widetilde{\chi }^{-}_{1}$. If the top squark is light enough to be on-shell and decays predominantly to a top quark and the LSP, gluinos decay through the chain $\widetilde{\mathrm{g}} \rightarrow {\widetilde{\mathrm{t}} _1} \overline{\mathrm{t}}\rightarrow \mathrm{t}\overline{\mathrm{t}} \widetilde{\chi }^{0}_{1}$ ($\mathrm {T}5\mathrm {tttt}$ model in Fig. 1, upper right). If instead the top squark mainly decays to the charm quark and the LSP, gluinos decay as in the $\mathrm {T}5\mathrm {ttcc}$ model (Fig. 1, lower left); in this case only two $\mathrm {W}$ bosons and two b jets are produced.

Events with four $\mathrm {W}$ bosons and two b jets can arise from bottom squark pair production, where each bottom squark decays to a top quark and a chargino, and the chargino decays to an LSP and a (possibly off-shell) $\mathrm {W}$ boson ($\mathrm {T}6\mathrm {tt}\mathrm {W}\mathrm {W}$ model in Fig. 1, lower middle).

Finally, SS lepton pairs can be produced in association with large values of $H_{\mathrm {T}}$, $E_{\mathrm {T}}^{\text {miss}}$, and $N_\text {jet}$, but without b jets. In particular, events with two $\mathrm {W}$ bosons and four light-flavor quark jets can arise from gluino pair production if each gluino decays to two light quarks and a chargino. The two charginos can have the same charge and each decay to a $\mathrm {W}$ boson and the LSP ($\mathrm {T}5\mathrm {qqqq}\mathrm {W}\mathrm {W}$ model in Fig. 1, lower right). In the case that the difference in mass between the chargino and the LSP is small, the $\mathrm {W}$ bosons are off-shell and produce soft leptons.

To increase the sensitivity to new-physics scenarios, we categorize events based on their kinematic properties as follows. First, three exclusive lepton selections are defined:

- high–high (HH) selection: two SS leptons, each with $p_{\mathrm {T}} \ge 25\,\text {GeV}$;
- high–low (HL) selection: two SS leptons, one with $p_{\mathrm {T}} \ge 25\,\text {GeV}$ and the other with $10 \le p_{\mathrm {T}} < 25\,\text {GeV}$;
- low–low (LL) selection: two SS leptons, each with $10 \le p_{\mathrm {T}} < 25\,\text {GeV}$.

The high lepton $p_{\mathrm {T}}$ threshold suppresses the contribution from nonprompt leptons; hence the SM background in the HH region arises primarily from events with prompt SS leptons. The nonprompt lepton background is largely contained in the HL region, where the high-$p_{\mathrm {T}}$ lepton is typically prompt and the low-$p_{\mathrm {T}}$ lepton nonprompt. The LL region is characterized by a very small background since all processes where at least one lepton originates from an on-shell vector boson are suppressed by the low-$p_{\mathrm {T}}$ requirements, while events with two nonprompt leptons are suppressed by the kinematic requirements described below; the main residual contribution in this region is from nonprompt leptons.

Second, search regions (SR) are introduced so that the analysis is sensitive to a variety of new-physics scenarios. SRs are defined separately for the HH, HL, and LL selections using the $H_{\mathrm {T}}$, $E_{\mathrm {T}}^{\text {miss}}$, $N_\text {jet}$, and $N_{\mathrm{b}}$ variables: $N_\text {jet}$ and $N_{\mathrm{b}}$ separate signal from background for scenarios with a large production of jets and/or b jets, while $H_{\mathrm {T}}$ and $E_{\mathrm {T}}^{\text {miss}}$ increase sensitivity to models with different masses of SUSY particles. In addition, we make use of the $M_\mathrm {T}^{\text {min}}$ variable, defined as:

5 $$\begin{aligned} M_\mathrm {T}^{\text {min}} = \min \left[ M_\mathrm {T} (\ell _1,E_{\mathrm {T}}^{\text {miss}}),M_\mathrm {T} (\ell _2,E_{\mathrm {T}}^{\text {miss}})\right] , \end{aligned}$$

where $M_\mathrm {T} (\ell ,E_{\mathrm {T}}^{\text {miss}}) = \sqrt{{2p_{\mathrm {T}} (\ell )E_{\mathrm {T}}^{\text {miss}} (1-\cos \phi _{\ell ,E_{\mathrm {T}}^{\text {miss}}})}}$ is the transverse mass and $\phi _{\ell ,E_{\mathrm {T}}^{\text {miss}}}$ is the azimuthal angle difference between the directions of the lepton and of the missing transverse momentum [48]. In the case of an SS lepton pair from $\mathrm{t}\overline{\mathrm{t}}$ or $\mathrm {W}$+jets processes, where one lepton is prompt and the other nonprompt, this variable has a cutoff near the $\mathrm {W}$ boson mass; consequently, the nonprompt lepton background is suppressed for SRs requiring $M_\mathrm {T}^{\text {min}} > 120\,\text {GeV}$ and is large for $M_\mathrm {T}^{\text {min}} < 120\,\text {GeV}$. In order to better characterize the background we use a fine SR binning in kinematic regions where SM processes are abundant (e.g., low $M_\mathrm {T}^{\text {min}}$ and low $E_{\mathrm {T}}^{\text {miss}}$), while, due to the low background, we use a coarser binning in regions with tight selections.

Finally, inclusive search regions in the HH and HL categories are defined in the tails of the $E_{\mathrm {T}}^{\text {miss}}$ and $H_{\mathrm {T}}$ variables; the boundaries $E_{\mathrm {T}}^{\text {miss}} >300\,\text {GeV}$ and $H_{\mathrm {T}} >1125\,\text {GeV}$ (for $E_{\mathrm {T}}^{\text {miss}} \le 300\,\text {GeV}$) are chosen so that each of these regions typically contains 1 background event.

A summary of the selection criteria is presented in Tables 2, 3 and 4. All SRs are non-overlapping. They are combined statistically to obtain the final results (Sect. 7).

**Table 2:** SR definitions for the HH selection. The notation $^\mathrm {(*)}$ indicates that, in order to avoid overlaps with SR31, an upper bound $E_{\mathrm {T}}^{\text {miss}} < 300\,\text {GeV}$ is used for regions with $H_{\mathrm {T}} > 300\,\text {GeV}$. All unlabeled region are included in the SR above them, for example the unlabeled regions between SR3 and SR9 are included in SR3, with the exception of the region to the right of SR31, which is included in SR31

$N_{\mathrm{b}}$	$M_\mathrm {T}^{\text {min}}$ ($\text {GeV}$)	$E_{\mathrm {T}}^{\text {miss}}$ ($\text {GeV}$)	$N_{\mathrm{b}}$	$H_{\mathrm {T}} < 300\,\text {GeV}$	$H_{\mathrm {T}} \in [300, 1125]\,\text {GeV}$	$H_{\mathrm {T}} > 1125\,\text {GeV}$
0	${<}120$	50–200	2–4	SR1	SR2	SR32
			${\ge }5$	SR3	SR4	
		${>}200^\mathrm {(*)}$	2–4		SR5	
			${\ge }5$		SR6	
	${>}120$	50–200	2–4		SR7	
			${\ge }5$		SR8	
		${>}200^\mathrm {(*)}$	2–4			
			$${\ge }5$$			
1	${<}120$	50–200	2–4	SR9	SR10	
			${\ge }5$	SR11	SR12	
		${>}200^\mathrm {(*)}$	2–4		SR13	
			${\ge }5$		SR14	
	${>}120$	50–200	2–4		SR15	
			${\ge }5$		SR16	
		${>}200^\mathrm {(*)}$	2–4			
			$${\ge }5$$			
2	${<}120$	50–200	2–4	SR17	SR18	
			${\ge }5$	SR19	SR20	
		${>}200^\mathrm {(*)}$	2–4		SR21	
			${\ge }5$		SR22	
	${>}120$	50–200	2–4		SR23	
			${\ge }5$		SR24	
		${>}200^\mathrm {(*)}$	2–4			
			$${\ge }5$$			
${\ge }3$	${<}120$	50–200	${\ge }2$	SR25	SR26	
		${>}200^\mathrm {(*)}$	${\ge }2$	SR27	SR28	
	${>}120$	${>}50^\mathrm {(*)}$	${\ge }2$	SR29	SR30	
Inclusive	Inclusive	${>}300$	${\ge }2$	–	SR31	

**Table 3:** SR definitions for the HL selection. The notation $^\mathrm {(*)}$ indicates that, in order to avoid overlaps with SR25, an upper bound $E_{\mathrm {T}}^{\text {miss}} < 300\,\text {GeV}$ is used for regions with $H_{\mathrm {T}} > 300\,\text {GeV}$. All unlabeled region are included in the SR above them, for example the unlabeled regions between SR3 and SR7 are included in SR3, with the exception of the region to the right of SR25, which is included in SR25

$N_{\mathrm{b}}$	$M_\mathrm {T}^{\text {min}}$ ($\text {GeV}$)	$E_{\mathrm {T}}^{\text {miss}}$ ($\text {GeV}$)	$N_{\mathrm{b}}$	$H_{\mathrm {T}} < 300\,\text {GeV}$	$H_{\mathrm {T}} \in [300, 1125]\,\text {GeV}$	$H_{\mathrm {T}} > 1125\,\text {GeV}$
0	${<}120$	50–200	2–4	SR1	SR2	SR26
			${\ge }5$	SR3	SR4	
		${>}200^\mathrm {(*)}$	2–4		SR5	
			${\ge }5$		SR6	
1	${<}120$	50–200	2–4	SR7	SR8	
			${\ge }5$	SR9	SR10	
		${>}200^\mathrm {(*)}$	2–4		SR11	
			${\ge }5$		SR12	
2	${<}120$	50–200	2–4	SR13	SR14	
			${\ge }5$	SR15	SR16	
		${>}200^\mathrm {(*)}$	2–4		SR17	
			${\ge }5$		SR18	
${\ge }3$	${<}120$	50–200	${\ge }2$	SR19	SR20	
		${>}200^\mathrm {(*)}$	${\ge }2$	SR21	SR22	
Inclusive	${>}120$	${>}50^\mathrm {(*)}$	${\ge }2$	SR23	SR24	
Inclusive	Inclusive	${>}300$	${\ge }2$	–	SR25	

**Table 4:** SR definitions for the LL selection. All SRs in this category require $N_{\mathrm{b}} \ge 2$. Unlabeled region to the right of SR7 and SR8 are included in SR7 and SR8, respectively

$N_{\mathrm{b}}$	$M_\mathrm {T}^{\text {min}}$ ($\text {GeV}$)	$H_{\mathrm {T}}$ ($\text {GeV}$)	$E_{\mathrm {T}}^{\text {miss}} \in [50, 200]\,\text {GeV}$	$E_{\mathrm {T}}^{\text {miss}} > 200\,\text {GeV}$
0	${<}120$	${>}300$	SR1	SR2
1			SR3	SR4
2			SR5	SR6
${\ge }3$			SR7	
Inclusive	${>}120$		SR8	

## Backgrounds

Backgrounds in the SS dilepton final state can be divided into three categories:

- **Nonprompt leptons:** Nonprompt leptons are leptons from heavy-flavor decays, hadrons misidentified as leptons, muons from light-meson decays in flight, or electrons from unidentified conversions of photons in jets. Depending on the signal region, this background is dominated by $\mathrm{t}\overline{\mathrm{t}}$ and $\mathrm {W}$+jets processes; it represents the largest background for regions with low $M_\mathrm {T}^{\text {min}}$ and low $H_{\mathrm {T}}$.
- **SM processes with SS dileptons:** Standard model processes that yield an SS lepton pair include multi-boson production (considering $\mathrm {W}$, $\mathrm{Z}$, $\mathrm{H}$, and prompt $\gamma$), single boson production in association with a $\mathrm{t}\overline{\mathrm{t}}$ pair, and double-parton scattering. The dominant sources are $\mathrm {W}\mathrm{Z}$ and $\mathrm{t}\overline{\mathrm{t}} \mathrm {W}$ production, which contribute primarily to SRs with zero and ${\ge }1$ b jets, respectively. $\mathrm {W}\mathrm{Z}$ events contribute to the background when the $\mathrm{Z}$ boson decays leptonically and is off-shell, when one of the $\mathrm{Z}$-boson decay leptons is not identified, or when the $\mathrm{Z}$ boson decays to $\tau$ leptons that result in a semileptonic final state. SM processes with SS dileptons are the largest background in the signal regions defined by tight kinematic selections.
- **Charge misidentification:** Charge misidentification arises from events with opposite-sign isolated leptons in which the charge of an electron is misidentified, mostly due to severe bremsstrahlung in the tracker material. Overall, this is a small background.

The nonprompt lepton background is estimated from data using the “tight-to-loose” ratio method, which was employed in previous versions of the analysis [19–23] but has been improved for the current study. It is based on a control sample of events (application region) where one lepton fails the nominal (tight) selection but passes the loose requirements, defined by relaxing the isolation selection for muons, and both the isolation and identification requirements for electrons. Events in this control region are reweighted by the factor ${\epsilon _{\mathrm {TL}}/(1-\epsilon _{\mathrm {TL}})}$, where $\epsilon _{\mathrm {TL}}$ is the probability for a nonprompt lepton that satisfies the loose selection to also satisfy the tight selection [19]. Its value is measured in a multijet-enriched data set (measurement region), using events from single-lepton triggers after applying a selection designed to suppress electroweak processes (Drell–Yan and $\mathrm {W}$+jets) and after subtracting their residual contribution; this selection requires only one lepton in the event, $E_{\mathrm {T}}^{\text {miss}} <20\,\text {GeV}$, and $M_\mathrm {T} <20\,\text {GeV}$. The measurement is made as a function of the lepton $p_{\mathrm {T}}$ and $\eta$, separately for each lepton flavor ($\mu$ or $\mathrm {e}$) and trigger (with or without isolation).

The method assumes that $\epsilon _{\mathrm {TL}}$ has the same value in the measurement and application regions. The main sources of discrepancy are identified as differences in the momentum spectrum and the flavor of the parton producing the nonprompt lepton. These two effects are mitigated in the following way. First, $\epsilon _{\mathrm {TL}}$ is parameterized as a function of $p_{\mathrm {T}} ^\text {corr}$, defined as the lepton $p_{\mathrm {T}}$ plus the energy in the isolation cone exceeding the isolation threshold value – this quantity is highly correlated with the mother parton $p_{\mathrm {T}}$, and thus the parameterization is robust against mother parton $p_{\mathrm {T}}$ variations. The second effect, i.e., flavor dependence, is relevant for electrons only: while nonprompt muons originate predominantly from heavy-flavor decays, nonprompt electrons receive sizable contributions from misidentified hadrons and conversions. The effect of variations in the flavor composition is suppressed by adjusting the loose electron identification criteria so that the numerical value of $\epsilon _{\mathrm {TL}}$ for electrons from light flavors matches that for electrons from heavy flavors. The loose lepton selection is defined based on MC studies, but we verify that $\epsilon _{\mathrm {TL}}$ is not significantly different in data events with and without b jets.

As a cross-check of the prediction, an alternative $\epsilon _{\mathrm {TL}}$ measurement, similar to that described in Ref. [49], is performed in the dilepton control region where one of the leptons fails the impact parameter requirement. The predictions from the two methods are found to be consistent, both in MC samples and in data.

The background from SM processes with a prompt SS lepton pair is evaluated from simulation, accounting for both theoretical and experimental uncertainties. The $\mathrm {W}\mathrm{Z}$ background is normalized to data in a control region requiring at least two jets, no b jets, $E_{\mathrm {T}}^{\text {miss}} >30\,\text {GeV}$, and three leptons, where two of the leptons form a same-flavor, opposite-sign pair with an invariant mass within 15$\,\text {GeV}$ of the Z boson mass; the measured normalization factor is found to be compatible with unity within about one standard deviation. The MC simulation of $\mathrm {W}\mathrm{Z}$ production is used to relate the number of expected WZ events in the signal regions to the WZ event yield in the control region.

Finally, the charge misidentification background is estimated by reweighting events with opposite-sign lepton pairs by the charge misidentification probability. For electrons this probability is obtained from simulated $\mathrm{t}\overline{\mathrm{t}}$ events and from ${\mathrm {e}^\pm \mathrm {e}^\pm }$ data in the Z mass window, and it lies in the range 10$^{-5}$–10$^{-3}$ depending on the electron $p_{\mathrm {T}}$ and $\eta$. Studies of simulated events indicate that the muon charge misidentification probability is negligible.

## Systematic uncertainties

Systematic uncertainties can affect both the overall normalization and the relative population of signal and background processes. A summary of their effects on the SR yields is given in Table 5.

**Table 5:** Summary of systematic uncertainties in the event yields in the SRs. The first six uncertainties are related to experimental factors for all processes whose yield is estimated from simulation; the next five are uncertainties in these yields related to the event simulation process itself. The last three uncertainties are related to background processes whose yield is estimated from data

Source	Typical uncertainty (%)
Lepton selection	2
Trigger efficiency	4
Jet energy scale	2–10
$\mathrm{b}$ tagging	5
Pileup	1–5
Integrated luminosity	2.7
Scale variations ($\mathrm{t}\overline{\mathrm{t}} \mathrm{Z}$ and $\mathrm{t}\overline{\mathrm{t}} \mathrm {W}$)	11–13
Parton distribution functions ($\mathrm{t}\overline{\mathrm{t}} \mathrm {W}$ and $\mathrm{t}\overline{\mathrm{t}} \mathrm{Z}$)	4
$\mathrm {W}^{\pm }\mathrm {W}^{\pm }$ normalization	30
Other backgrounds	50
Monte Carlo statistical precision	1–30
Nonprompt leptons	30–36
Charge misidentification	26
$\mathrm {W}\mathrm{Z}$ normalization	30

**Fig. 2:**
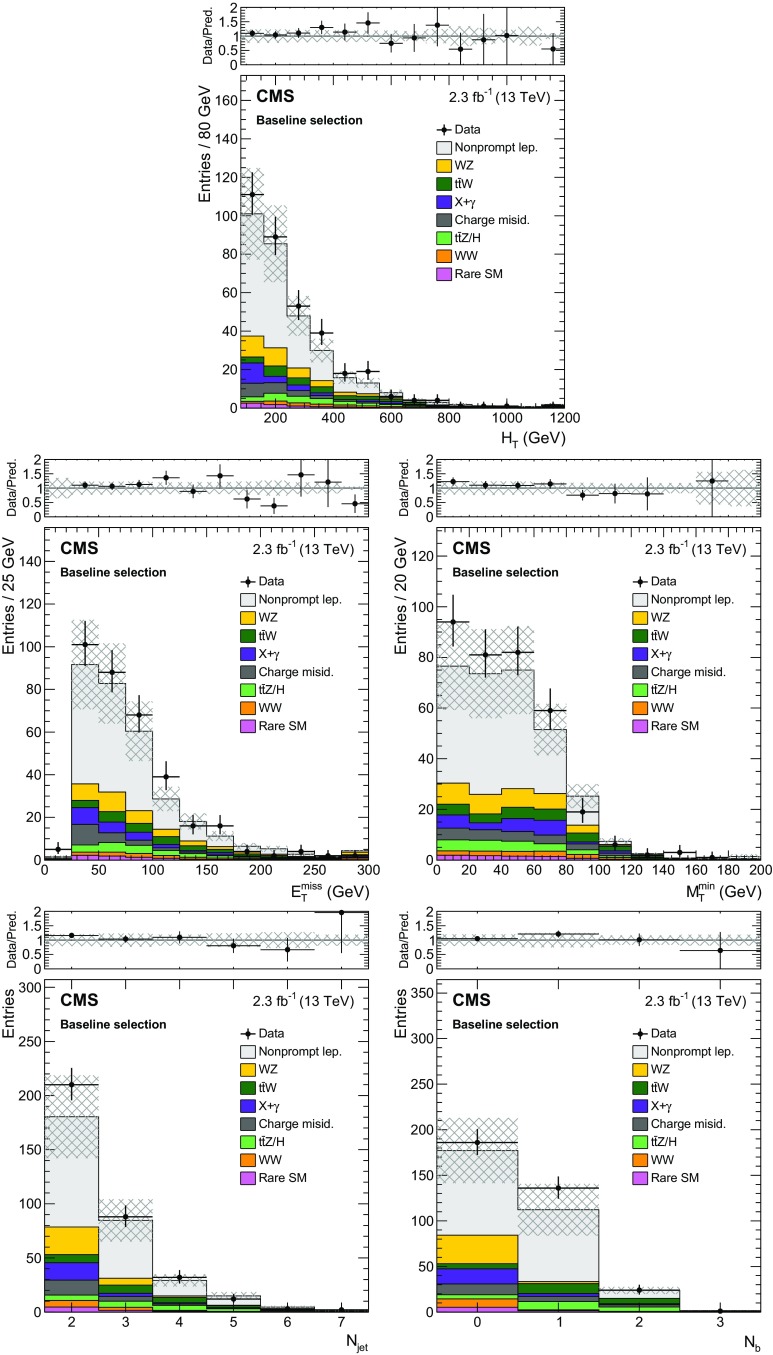
Distributions of the main analysis variables: $H_{\mathrm {T}}$  (*top*), $E_{\mathrm {T}}^{\text {miss}}$  (*middle left*), $M_\mathrm {T}^{\text {min}}$  (*middle right*), $N_\text {jet}$  (*bottom left*), and $N_{\mathrm{b}}$  (*bottom right*), after a baseline selection requiring a pair of SS leptons, two jets, and either $E_{\mathrm {T}}^{\text {miss}} > 30\,\text {GeV}$ or $H_{\mathrm {T}} > 500\,\text {GeV}$. The last bin includes the overflow. The notation X+$\gamma$ refers to processes with a prompt photon in the final state. The *hatched area* represents the total uncertainty in the background prediction. The *upper panels* show the ratio of the observed event yield to the background prediction

**Fig. 3:**
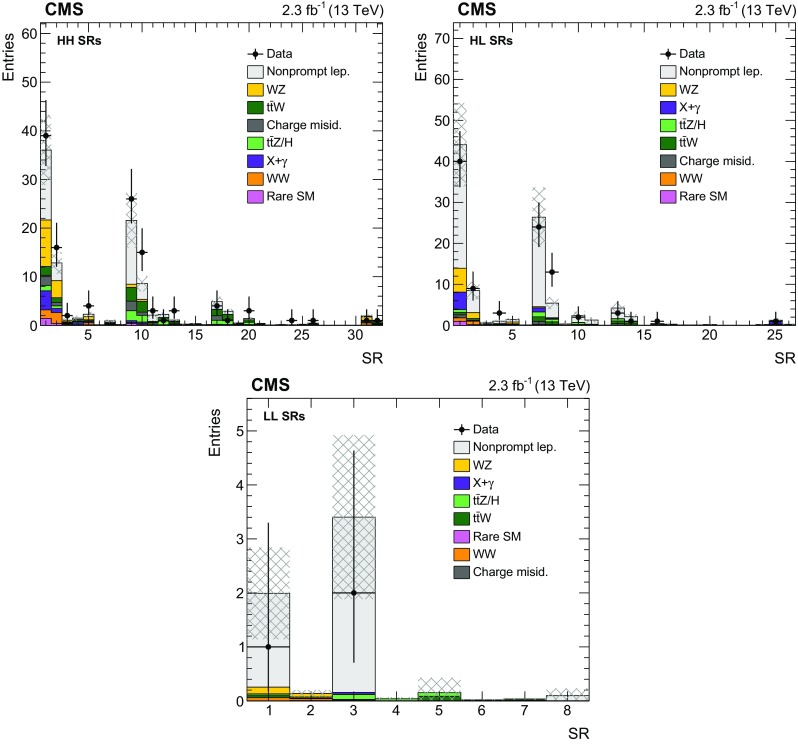
Event yields in the HH (*top left*), HL (*top right*), and LL (*bottom*) SRs. The notation X+$\gamma$ refers to processes with a prompt photon in the final state. The *hatched area* represents the total uncertainty in the background prediction

**Table 6:** Expected number of background and observed events for the different SRs considered in this analysis

Region	HH event yields		HL event yields		LL event yields	
	Expected SM	Observed	Expected SM	Observed	Expected SM	Observed
SR1	36.0 ± 7.0	39	44.1 ± 10.9	40	1.99 ± 0.94	1
SR2	12.8 ± 2.1	16	8.5 ± 2.1	9	0.14 ± 0.07	0
SR3	1.05 ± 0.36	2	0.61 ± 0.36	0	3.4 ± 1.5	2
SR4	1.49 ± 0.52	0	1.01 ± 0.38	3	0.04 ± 0.03	0
SR5	2.29 ± 0.49	4	1.40 ± 0.37	0	0.15 ± 0.28	0
SR6	0.11 ± 0.04	0	0.08 ± 0.04	0	0.02 ± 0.01	0
SR7	0.91 ± 0.31	0	26.4 ± 7.6	24	0.03 ± 0.01	0
SR8	0.16 ± 0.06	0	5.4 ± 1.5	13	0.10 ± 0.10	0
SR9	21.6 ± 5.2	26	0.34 ± 0.20	0		
SR10	8.6 ± 1.4	15	2.37 ± 0.99	2		
SR11	2.10 ± 0.92	3	1.29 ± 0.65	0		
SR12	2.24 ± 0.40	1	0.05 ± 0.04	0		
SR13	1.09 ± 0.21	3	4.2 ± 1.3	3		
SR14	0.25 ± 0.11	0	2.11 ± 0.69	1		
SR15	0.37 ± 0.12	0	0.06 ± 0.03	0		
SR16	0.19 ± 0.08	0	0.42 ± 0.09	1		
SR17	4.9 ± 1.0	4	0.29 ± 0.15	0		
SR18	2.90 ± 0.47	1	0.10 ± 0.08	0		
SR19	0.47 ± 0.09	0	0.11 ± 0.06	0		
SR20	1.43 ± 0.25	3	0.18 ± 0.17	0		
SR21	0.40 ± 0.10	0	0.001 ± 0.001	0		
SR22	0.08 ± 0.04	0	0.04 ± 0.04	0		
SR23	0.17 ± 0.06	0	0.03 ± 0.03	0		
SR24	0.14 ± 0.04	1	0.21 ± 0.17	0		
SR25	0.21 ± 0.06	0	1.25 ± 0.53	1		
SR26	0.46 ± 0.12	1	0.25 ± 0.12	0		
SR27	0.005 ± 0.016	0				
SR28	0.03 ± 0.02	0				
SR29	0.02 ± 0.01	0				
SR30	0.02 ± 0.01	0				
SR31	1.91 ± 0.32	1				
SR32	0.85 ± 0.18	1				

Experimental systematic uncertainties are mostly the consequence of differing event selection efficiencies in data and simulation. Lepton identification and trigger efficiencies are computed with the “tag-and-probe” technique [26, 27] with an uncertainty of 2 and 4 %, respectively. For signal samples, additional uncertainties of 4–10 % are included to account for differences in the lepton efficiency between the fast and Geant4-based simulations. The jet energy scale uncertainty varies between 2 and 8 %, depending on the jet $p_{\mathrm {T}}$ and $\eta$. Its impact is assessed by shifting the energy of each jet and propagating the variation to all dependent kinematic quantities ($H_{\mathrm {T}}$, $E_{\mathrm {T}}^{\text {miss}}$, $N_\text {jet}$, $N_{\mathrm{b}}$, and $M_\mathrm {T}^{\text {min}}$); correlation effects due to the migration of events from one SR to another are taken into account. These variations yield estimated uncertainties of 2–10 %. A similar approach is used to estimate the uncertainties associated with the $\mathrm{b}$ tagging efficiencies for light-flavor and b quark jets [36], which are parameterized as a function of $p_{\mathrm {T}}$ and $\eta$ and are found to be of order 5 % for the highly populated SRs. The uncertainty in the modeling of pileup is 1–5 % depending on the SR. The uncertainty in the integrated luminosity is 2.7 % [50].

**Fig. 4:**
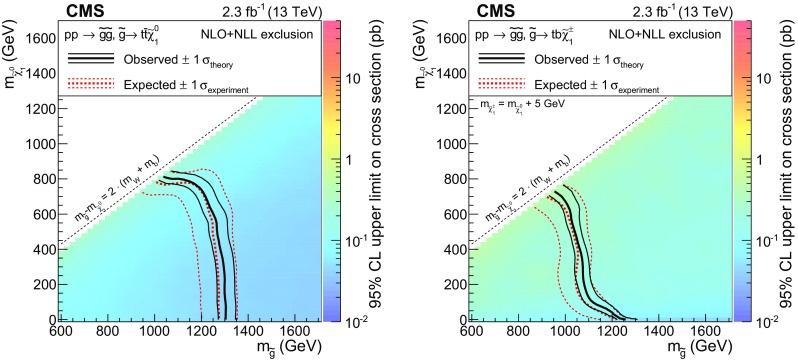
Exclusion regions at the 95 % CL in the $m_{\widetilde{\chi }^{0}_{1}}$ versus $m_{\widetilde{\mathrm{g}}}$ plane for the $\mathrm {T}1\mathrm {tttt}$  (*left*) and $\mathrm {T}5\mathrm {ttbb}\mathrm {W}\mathrm {W}$  (*right*) models, where for the $\mathrm {T}5\mathrm {ttbb}\mathrm {W}\mathrm {W}$ model $m_{\widetilde{\chi }^\pm _{1}} = m_{\widetilde{\chi }^{0}_{1}} + 5\,\text {GeV}$. The *right-hand side color scale* indicates the excluded cross section values for a given point in the SUSY particle mass plane. The *solid*, *black curves* represent the observed exclusion limits assuming the NLO+NLL cross sections (*thick line*), or their variations of ±1 standard deviation (*thin lines*). The *dashed*, *red curves* show the expected limits with the corresponding ±1 standard deviation experimental uncertainties. Excluded regions are to the *left* and *below the limit curves*

The background sources estimated from simulation are subject to theoretical uncertainties related to unknown higher-order effects and to uncertainties in the knowledge of the PDFs. The former are estimated by simultaneously varying the renormalization and factorization scales up and down by a factor of two. The effect on the overall cross section is found to be 13 % for $\mathrm{t}\overline{\mathrm{t}} \mathrm {W}$ events and 11 % for $\mathrm{t}\overline{\mathrm{t}} \mathrm{Z}$ events, while the effect on the acceptance for the various SRs amounts to 3–8 % depending on $H_{\mathrm {T}}$. The magnitude of the uncertainty related to the PDFs is obtained using variations of the NNPDF3.0 set [40]. The overall uncertainty is ${\sim } 4~\%$ for the $\mathrm{t}\overline{\mathrm{t}} \mathrm {W}$ and $\mathrm{t}\overline{\mathrm{t}} \mathrm{Z}$ samples. Theoretical uncertainties are also considered for the remaining minor backgrounds estimated from simulation: a similar procedure is used for the $\mathrm {W}^{\pm }\mathrm {W}^{\pm }$ process, leading to an overall uncertainty of 30 %, while a 50 % uncertainty is assigned to processes with a prompt $\gamma$ and to the sum of the other rare processes. For all backgrounds estimated from simulation we account for the statistical uncertainty of the MC samples.

The remaining sources of uncertainty are those related to the methods that are used to estimate the nonprompt lepton, charge misidentification, and $\mathrm {W}\mathrm{Z}$ backgrounds. An overall normalization uncertainty of 30 % is assigned to the nonprompt lepton background prediction. This uncertainty accounts for the performance of the method on simulated data and for the differences in the prediction from the two alternative procedures described in Sect. 5. An additional uncertainty is associated with the subtraction procedure to remove Drell–Yan and $\mathrm {W}$+jets events from the measurement region; the overall effect on the nonprompt lepton background yield is 1–20 %, depending on the SR considered, and is larger for high-$p_{\mathrm {T}}$ leptons. Finally, we account for the statistical uncertainty in the number of events observed in the application region.

The background from charge misidentification is assigned a systematic uncertainty of 26 %, which corresponds to the difference between the ${\mathrm {e}^\pm \mathrm {e}^\pm }$ event yield in the Z mass window in data and simulation.

The uncertainty in the $\mathrm {W}\mathrm{Z}$ background is measured to be 30 % in the control region. It includes statistical uncertainties and systematic uncertainties due to non-$\mathrm {W}\mathrm{Z}$ background subtraction. Using the same procedure as described above, uncertainties in the extrapolation from the control to the signal regions are assessed from the propagation of the uncertainty in the jet energy scale and in the $\mathrm{b}$ tagging efficiencies.

**Fig. 5:**
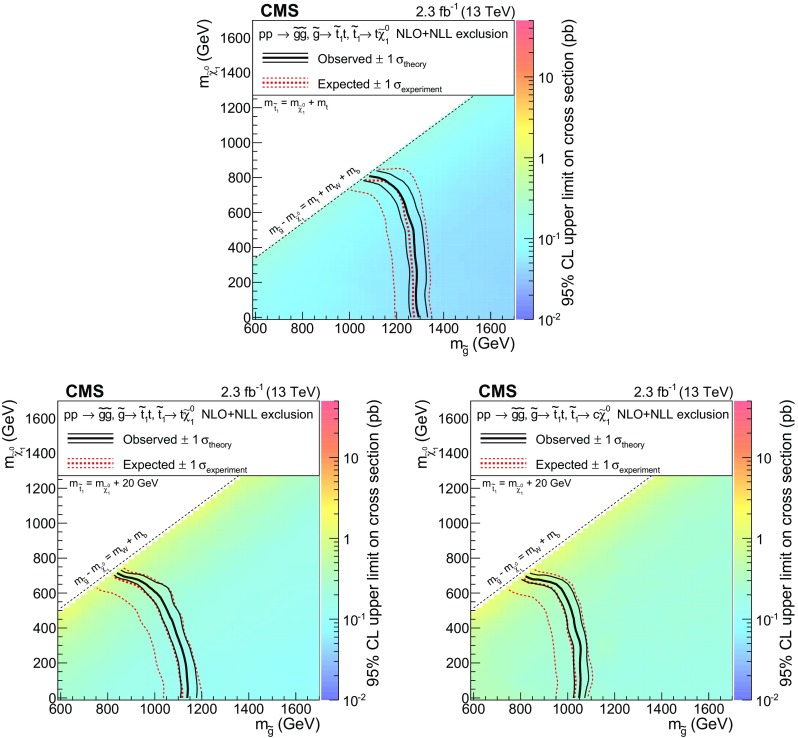
Exclusion regions at the 95 % CL in the plane of $m_{\widetilde{\chi }^{0}_{1}}$ versus $m_{\widetilde{\mathrm{g}}}$ for models with the gluino decaying to an on-shell top squark: $\mathrm {T}5\mathrm {tttt}$ with $m_{{\widetilde{\mathrm{t}} _1}} = m_{\widetilde{\chi }^{0}_{1}} + m_{\mathrm{t}}$ (*top*), $\mathrm {T}5\mathrm {tttt}$ with $m_{{\widetilde{\mathrm{t}} _1}} = m_{\widetilde{\chi }^{0}_{1}} + 20\,\text {GeV}$ (*bottom left*), and $\mathrm {T}5\mathrm {ttcc}$ with $m_{{\widetilde{\mathrm{t}} _1}} = m_{\widetilde{\chi }^{0}_{1}} + 20\,\text {GeV}$ (*bottom right*). For a description of the notation, see Fig. 4

## Results

Distributions of the five kinematic variables used to define the SRs, $H_{\mathrm {T}}$, $E_{\mathrm {T}}^{\text {miss}}$, $M_\mathrm {T}^{\text {min}}$, $N_\text {jet}$, and $N_{\mathrm{b}}$, are shown in Fig. 2 after a baseline selection requiring a pair of SS leptons, two jets, and either $E_{\mathrm {T}}^{\text {miss}} > 30\,\text {GeV}$ or $H_{\mathrm {T}} > 500\,\text {GeV}$. The results are shown in comparison to the background prediction. The event yields in the SRs after the full selection are presented in Fig. 3 and in Table 6; no significant deviation from the SM background prediction is observed. The largest local significances are 2.2 and 1.8 standard deviations in HL SR8 and in HH SR10, respectively.

The results of the search are used to constrain the benchmark SUSY models presented in Sect. 4. For each mass point in the SUSY particle mass spectrum, results from all SRs are combined to extract cross section exclusion limits at the 95 % confidence level (CL), using the asymptotic formulation of the modified frequentist CL$_\mathrm {s}$ criterion [51–54]. Signal and background uncertainties are included as log-normal nuisance parameters and, when relevant, take into account correlation effects among different SRs and/or different processes. Exclusion contours make use of the cross section values calculated at NLO plus next-to-leading logarithmic (NLL) accuracy, assuming that all SUSY particles other than those included in the respective diagram are too heavy to participate in the interaction [55–60]. In general, the SR with the largest sensitivity is HH SR31, which requires $E_{\mathrm {T}}^{\text {miss}} >300\,\text {GeV}$ and is inclusive in the other variables. However, depending on the model and the region of parameter space, other SRs contribute significantly to the total sensitivity: for instance, a considerable contribution comes from HL SR25 in case of signal models with a soft lepton, from HH SR32 and HL SR26 in case of high $H_{\mathrm {T}}$, from HH SR3 and SR8 in case of no b jets, and from HH SR24 and SR26 in case of 2 or more b jets.

Results for models with gluinos decaying to virtual third generation squarks are shown in Fig. 4 as a function of the gluino and LSP masses. For the $\mathrm {T}1\mathrm {tttt}$ model (Fig. 4-left), in the regions of the SUSY parameter space with a large mass difference between the gluino and the LSP, the results are rather stable with respect to LSP mass variations, and gluino masses up to 1300$\,\text {GeV}$ are excluded. Near the kinematic threshold $m_{\widetilde{\mathrm{g}}}-m_{\widetilde{\chi }^{0}_{1}} = 2(m_{\mathrm {W}}+m_{\mathrm{b}})$, the gluino mass limit becomes weaker and is reduced to 1050$\,\text {GeV}$ for an LSP mass of 800$\,\text {GeV}$. Results for the $\mathrm {T}5\mathrm {ttbb}\mathrm {W}\mathrm {W}$ model with nearly degenerate $\widetilde{\chi }^\pm _{1}$ and $\widetilde{\chi }^{0}_{1}$ masses are shown in Fig. 4-right; the limit on the gluino mass lies in the range 950–1100$\,\text {GeV}$ except for very small $\widetilde{\chi }^\pm _{1}$ and $\widetilde{\chi }^{0}_{1}$ masses, where the sensitivity increases because of the large Lorentz boost of the leptons from the $\widetilde{\chi }^\pm _{1}$ decay.

**Fig. 6:**
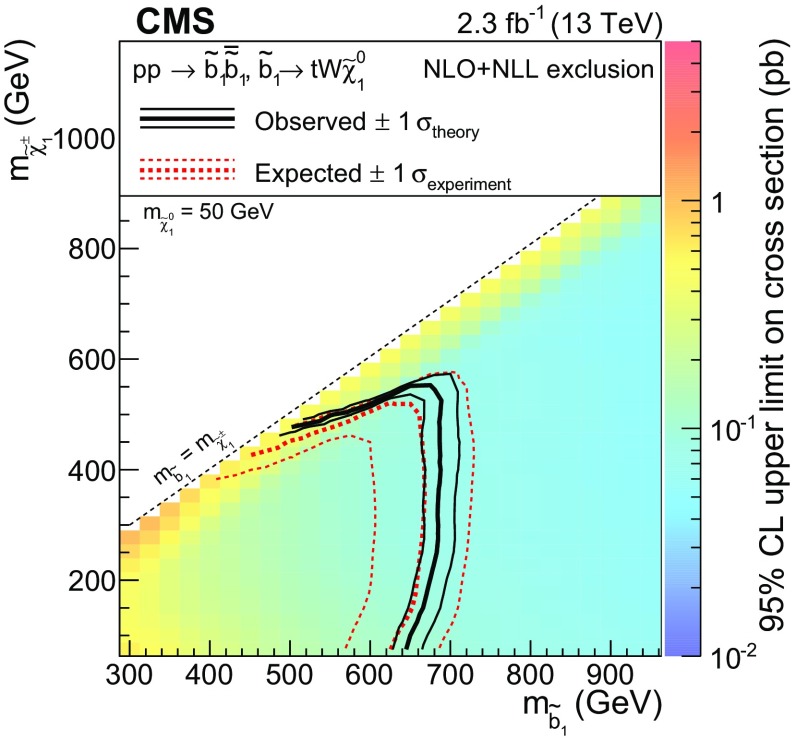
Exclusion regions at the 95 % CL in the plane of $m_{\widetilde{\chi }^\pm _{1}}$ versus $m_{{\widetilde{\mathrm{b}} _1}}$ for the $\mathrm {T}6\mathrm {tt}\mathrm {W}\mathrm {W}$ model with $m_{\widetilde{\chi }^{0}_{1}}=50\,\text {GeV}$. For a description of the notation, see Fig. 4

Results for models with a gluino decaying to an on-shell top squark are shown in Fig. 5 as a function of the gluino and LSP masses. For the $\mathrm {T}5\mathrm {tttt}$ model (Fig. 5-top), for which we take $m_{{\widetilde{\mathrm{t}} _1}} = m_{\widetilde{\chi }^{0}_{1}} + m_{\mathrm{t}}$, similar exclusion curves are obtained as for the T1tttt model in Fig. 4-left because the production cross section and the final-state particles are the same. The limit becomes weaker when there is a small mass difference between the top squark and the LSP: for $m_{{\widetilde{\mathrm{t}} _1}} - m_{\widetilde{\chi }^{0}_{1}} = 20\,\text {GeV}$, the limit on the gluino mass is 1140$\,\text {GeV}$ for small LSP masses and about 850$\,\text {GeV}$ for $m_{\widetilde{\chi }^{0}_{1}} = 700\,\text {GeV}$ (Fig. 5-bottom left). In the case of the $\mathrm {T}5\mathrm {ttcc}$ model with the same SUSY particle mass values, the sensitivity is slightly reduced because of the smaller number of leptons and b jets in the final state (Fig. 5-bottom right).

Figure 6 shows the results for b squark production in the $\mathrm {T}6\mathrm {tt}\mathrm {W}\mathrm {W}$ model in the chargino ($\widetilde{\chi }^\pm _{1}$) versus b squark mass plane, where the LSP mass is assumed to be $m_{\widetilde{\chi }^{0}_{1}}=50\,\text {GeV}$. For chargino masses up to 550$\,\text {GeV}$, b squark masses below 680$\,\text {GeV}$ are excluded. The limit on the b squark mass is reduced to 500$\,\text {GeV}$ in regions where $m_{\widetilde{\chi }^\pm _{1}}$ is within 100$\,\text {GeV}$ of $m_{{\widetilde{\mathrm{b}} _1}}$, while a milder reduction is observed in regions where the difference between $m_{\widetilde{\chi }^\pm _{1}}$ and $m_{\widetilde{\chi }^{0}_{1}}$ is less than 150$\,\text {GeV}$.

**Fig. 7:**
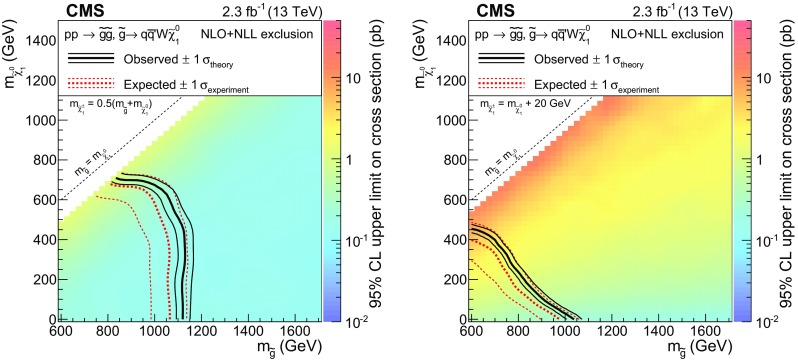
Exclusion regions at the 95 % CL in the plane of $m_{\widetilde{\chi }^{0}_{1}}$ versus $m_{\widetilde{\mathrm{g}}}$ for the $\mathrm {T}5\mathrm {qqqq}\mathrm {W}\mathrm {W}$ model with $m_{\widetilde{\chi }^\pm _{1}}=0.5(m_{\widetilde{\mathrm{g}}} + m_{\widetilde{\chi }^{0}_{1}})$ (*left*) and with $m_{\widetilde{\chi }^\pm _{1}} = m_{\widetilde{\chi }^{0}_{1}} + 20\,\text {GeV}$ (*right*). For a description of the notation, see Fig. 4

Results for the $\mathrm {T}5\mathrm {qqqq}\mathrm {W}\mathrm {W}$ model are shown in Fig. 7 as a function of the gluino and LSP masses, with two different assumptions for the chargino mass: it is either assumed to be the average of $m_{\widetilde{\mathrm{g}}}$ and $m_{\widetilde{\chi }^{0}_{1}}$, or it is set to $m_{\widetilde{\chi }^{0}_{1}} + 20\,\text {GeV}$. In the first case (Fig. 7-left), the exclusion limit on gluino masses exceeds 1100$\,\text {GeV}$ for LSP masses up to 400$\,\text {GeV}$; for larger LSP masses the limit is reduced to 830$\,\text {GeV}$ at $m_{\widetilde{\chi }^{0}_{1}} = 700\,\text {GeV}$. In the second case (Fig. 7-right), due to the smaller mass difference, leptons in the final state are soft and thus the sensitivity is reduced.

The results of the search are also used to set 95 % CL upper limits on the double $\mathrm{t}\overline{\mathrm{t}}$ production cross section, whose SM value computed at NLO precision [37] is 9.1$\,\text {fb}$. The upper limit on $\sigma ({\mathrm {p}\mathrm {p}} \rightarrow {\mathrm{t}\overline{\mathrm{t}} \mathrm{t}\overline{\mathrm{t}}})$ is found to be 119$\,\text {fb}$, with an expected result of $102^{+57}_{-35}\,\text {fb}$. With the current integrated luminosity, the sensitivity to this signature is limited by the statistical precision.

Limits at the 95 % CL on the SS top quark pair production cross section are determined using events that satisfy the baseline selection categorized according to number of b jets (Fig. 2-bottom right); apart from the charge requirement, the detector acceptance and the selection efficiency for the signal are assumed to match those of SM $\mathrm{t}\overline{\mathrm{t}}$ events. The observed (expected) upper limit on $\sigma (\mathrm {p}\mathrm {p}\rightarrow {\mathrm{t} \mathrm{t}}) + \sigma ({\mathrm {p}\mathrm {p}} \rightarrow {\overline{\mathrm{t}} \overline{\mathrm{t}}})$ is 1.7$\,\text {pb}$  ($1.5^{+0.7}_{-0.4}\,\text {pb}$).

**Fig. 8:**
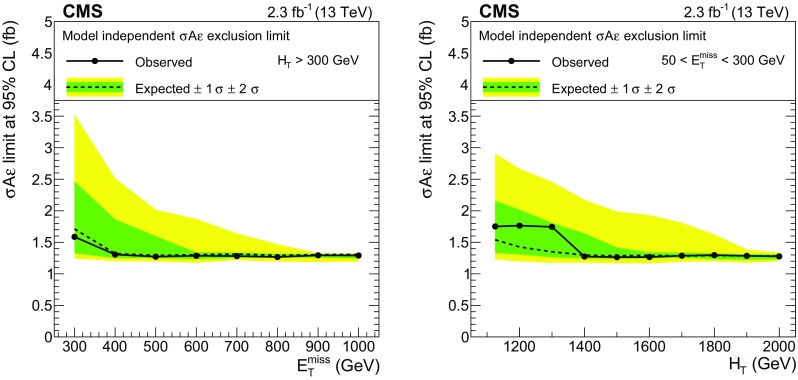
Limits on the product of cross section, detector acceptance, and selection efficiency, $\sigma \! \mathcal {A} \epsilon$, for the production of an SS dilepton pair as a function of $E_{\mathrm {T}}^{\text {miss}}$ in HH SR31 (*left*) and of $H_{\mathrm {T}}$ in HH SR32 (*right*)

Finally, we report model independent limits on the product of cross section, detector acceptance, and selection efficiency, $\sigma \! \mathcal {A} \epsilon$, for the production of an SS dilepton pair in the two inclusive HH regions, SR31 and SR32, using the CL$_\mathrm {s}$ criterion without the asymptotic approximation. In SR31 the limit is computed as a function of the minimum threshold on $E_{\mathrm {T}}^{\text {miss}}$ for $H_{\mathrm {T}} >300\,\text {GeV}$, while in SR32 it is computed as a function of the $H_{\mathrm {T}}$ threshold for $50<E_{\mathrm {T}}^{\text {miss}} <300\,\text {GeV}$. The results are shown in Fig. 8, where, in regions with no observed events, the minimum limit value of 1.3$\,\text {fb}$ is obtained. These limits can be used to test additional BSM models, after accounting for the event selection efficiency. The lepton efficiency ranges between 70–85 % (45–70 %) for generated muons (electrons) with $|\eta |<2.4$ and $p_{\mathrm {T}} >25\,\text {GeV}$, increasing as a function of $p_{\mathrm {T}}$ and converging to the maximum value for $p_{\mathrm {T}} >60\,\text {GeV}$; the efficiencies of the $H_{\mathrm {T}}$ and $E_{\mathrm {T}}^{\text {miss}}$ requirements are mostly determined by the jet energy and $E_{\mathrm {T}}^{\text {miss}}$ resolutions, which are discussed in Refs. [33, 35].

## Summary

The results of a search for new physics in same-sign dilepton events using the CMS detector at the LHC and based on a data sample of pp collisions at $\sqrt{s} = 13\,\text {TeV}$, corresponding to an integrated luminosity of 2.3$\,\text {fb}^{-1}$, are presented. The data are analyzed in nonoverlapping signal regions defined with different selections on lepton and event kinematic variables, as well as jet and b quark jet multiplicities.

No significant deviation from the standard model expectations is observed. The results are used to set limits on the production of supersymmetric particles in various simplified models. Gluino and bottom squark masses are excluded at the 95 % confidence level up to 1300 and 680$\,\text {GeV}$, respectively. These results extend the limits obtained in the previous version of the analysis [23] by about 250$\,\text {GeV}$ on the gluino mass, and 150$\,\text {GeV}$ on the bottom squark mass. In addition, 95 % confidence level upper limits of 119$\,\text {fb}$ and 1.7$\,\text {pb}$ are set on the cross sections for the production of two top quark-antiquark pairs and for the production of two SS top quarks, respectively. Model independent limits and selection efficiencies are provided to allow further interpretations of the results, using alternative models to those examined here.
